# General overview of the current status of human foodborne trematodiasis

**DOI:** 10.1017/S0031182022000725

**Published:** 2022-09

**Authors:** Jong-Yil Chai, Bong-Kwang Jung

**Affiliations:** 1 Department of Tropical Medicine and Parasitology, Seoul National University College of Medicine, Seoul 03080, South Korea; 2 MediCheck Research Institute, Korea Association of Health Promotion, Seoul 07649, South Korea

**Keywords:** Human foodborne trematode, intestinal fluke, liver fluke, lung fluke, paratenic host

## Abstract

Foodborne trematodes (FBT) of public health significance include liver flukes (*Clonorchis sinensis, Opisthorchis viverrini, O. felineus, Fasciola hepatica* and *F. gigantica*), lung flukes (*Paragonimus westermani* and several other *Paragonimus* spp.) and intestinal flukes, which include heterophyids (*Metagonimus yokogawai, Heterophyes nocens* and *Haplorchis taichui*), echinostomes (*Echinostoma revolutum, Isthmiophora hortensis, Echinochasmus japonicus* and *Artyfechinostomum malayanum*) and miscellaneous species, including *Fasciolopsis buski* and *Gymnophalloides seoi*. These trematode infections are distributed worldwide but occur most commonly in Asia. The global burden of FBT diseases has been estimated at about 80 million, however, this seems to be a considerable underestimate. Their life cycle involves a molluscan first intermediate host, and a second intermediate host, including freshwater fish, crustaceans, aquatic vegetables and freshwater or brackish water gastropods and bivalves. The mode of human infection is the consumption of the second intermediate host under raw or improperly cooked conditions. The major pathogenesis of *C. sinensis* and *Opisthorchis* spp. infection includes inflammation of the bile duct which leads to cholangitis and cholecystitis, and in a substantial number of patients, serious complications, such as liver cirrhosis and cholangiocarcinoma, may develop. In lung fluke infections, cough, bloody sputum and bronchiectasis are the most common clinical manifestations. However, lung flukes often migrate to extrapulmonary sites, including the brain, spinal cord, skin, subcutaneous tissues and abdominal organs. Intestinal flukes can induce inflammation in the intestinal mucosa, and they may at times undergo extraintestinal migration, in particular, in immunocompromised patients. In order to control FBT infections, eating foods after proper cooking is strongly recommended.

## Graphical abstract

**Figure:**
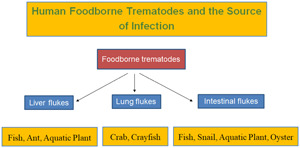

## Introduction

Foodborne trematodes (FBT) are defined as trematodes infecting humans, which are transmitted by consumption of foods, globally or locally available (including traditional foods) or those taken rarely or accidentally. In 1995, the World Health Organization (WHO) estimated the total global number of people infected with FBT at more than 40 million (WHO, 1995). A decade later (in 2005), about 56.2 million people were estimated to be infected with FBT, with 7.9 million having severe sequelae and 7158 people dying mostly from cholangiocarcinoma and cerebral infections (Fürst *et al*., 2012a). The most recent estimate extrapolated the total global number of people infected with FBT at 74.7 million as of 2015–2016 with 0.2 million new cases annually and 2 million disability-adjusted life years (Fürst *et al*., 2019; WHO, 2020). However, there are problems of low sensitivity and specificity of diagnostic tools as well as possible numerous endemic areas so far undetected, and these global estimates of the FBT burden seem to be much underestimated. Thus, the WHO road map for ‘control of FBT diseases 2021–2030’ recommends critical actions, including the development of accurate surveillance and mapping tools and methods, with information on environmental factors involved in infection, and the promotion of application and awareness of preventive chemotherapy (WHO, 2020).

Taxonomically, the FBTs are highly diverse, and at least 99 species (15 liver flukes, 9 lung flukes and 75 intestinal flukes) have been reported from human infections (Chai *et al*., 2005; Chai, 2007, 2019; Fürst *et al*., 2012a; Chai and Jung, 2019, 2020; Cho *et al*., 2020). Among them, those of greatest public health significance are *Clonorchis sinensis, Opisthorchis* spp., *Fasciola hepatica*, *Paragonimus westermani*, and several species of intestinal flukes, such as *Metagonimus* spp., *Haplorchis* spp. and echinostomes (WHO, 1995; Chai and Jung, 2019).

The origin of FBTs may date back to almost 8000 BCE when humans started to switch from a nomadic, hunter-gatherer lifestyle to a settled, agricultural way of life (Steverding, 2020). However, from the evolutionary point of view, intestinal flukes seem to be the oldest group, which subsequently evolved to parasitize the bile duct and liver (liver flukes) or lungs (lung flukes) in the human body.

The mode of human infection with FBT is closely linked to human behavioural patterns in endemic localities, specifically the methods of food production, preparation and consumption (WHO, 1995). Thus, the epidemiology of FBT infection is determined by ecological and environmental factors related to food and is strongly influenced by poverty, pollution and population growth (WHO, 1995). The infection source, i.e. food, is diverse, including aquatic or semi-terrestrial snails (gastropods and bivalves), fish, crustaceans, water plants, amphibians and reptiles (WHO, 1995; Chai, 2019). Rarely insects and mammals may serve as the source of human infection for some species (Chai, 2019; Chai and Jung, 2019).

The geographical distribution of FBT is almost worldwide. However, as FBT infections are closely related to food habits, they are predominantly found in East Asian and Western Pacific countries where local people prefer to eat various types of foods raw or under improperly cooked conditions (WHO, 1995). This kind of food habit is in most cases traditional and one of the longstanding customs, so it is very difficult to change within a short period of time. Health education targeting schoolchildren is an important strategy for long-term control of FBT infections. Control of infection sources, including intermediate hosts, is very difficult and practically almost impossible. Mass drug administration (MDA) using an effective anthelmintic, such as praziquantel, may be feasible to lower the infection rate of people in endemic communities. However, if reinfection persists, the prevalence would rise again to the previous level. Thus, control of FBT infections by infrequent MDA may be insufficient. Repeated MDA with sustained health education of young people in endemic communities is an ideal control strategy.

In this review, the authors briefly summarized the current status of FBT occurring around the world. The taxonomy and phylogeny of FBT, their life cycles, mode of transmission, epidemiology and geographical distribution, global disease burden, pathogenicity and clinical manifestations, diagnostic problems, anthelmintics used, and control strategies are briefly reviewed.

## Liver flukes

At least 15 species of liver flukes are known to cause human infections. They include *C. sinensis, Opisthorchis viverrini, O. felineus, Metorchis conjunctus, M. bilis, M. orientalis, Amphimerus* sp., *Amphimerus noverca, A. pseudofelineus, Pseudamphistomum truncatum, P. aethiopicum, Dicrocoelium dendriticum, D. hospes, Fasciola hepatica* and *F. gigantica* (Table 1) (Chai and Jung, 2019). They can be divided into small liver flukes (*C. sinensis, Opisthorchis* spp., *Metorchis* spp., *Amphimerus* spp., *Pseudamphistomum* spp. and *Dicrocoelium* spp.) and large liver flukes (*F. hepatica* and *F. gigantica*) according to the size of the adult worms. The first intermediate host is freshwater or brackish water snails, and the second intermediate host is freshwater fish (*C. sinensis, Opisthorchis* spp., *Metorchis* spp., *Amphimerus* spp. and *Pseudamphistomum* spp.), ants (*Dicrocoelium* spp.) or aquatic vegetation (*Fasciola* spp.) (Table 1). The morphological similarity of opisthorchiid eggs to heterophyid as well as lecithodendriid-like fluke eggs frequently poses diagnostic problems in human fecal examinations. Molecular techniques using internal transcribed spacers (ITS) and mitochondrial cytochrome *c* oxidase 1 (*cox*1) have been used to differentiate the eggs as well as larvae and adults of opisthorchiid and heterophyid flukes (Duflot *et al*., 2021).

**Table 1.** Liver flukes infecting humans with biological, clinical characteristics and geographical distribution

Group/Species of flukes	First intermediate host (snail)	Second intermediate host	Reservoir host	Major clinical characteristics	Geographical distribution^a^
*Clonorchis sinensis*	*Parafossarulus* sp.	Freshwater fish	Dog, cat, rat, pig, badger, weasel, camel, buffalo	Cholangitis, cirrhosis, cholangiocarcinoma	China, Taiwan, South Korea, Vietnam, Russia
*Opisthorchis viverrini*	*Bithynia* sp.	Freshwater fish	Dog, cat, rat, pig	Cholangitis, cirrhosis, cholangiocarcinoma	Thailand, Laos, Vietnam, Cambodia, Malaysia, Myanmar
*Opisthorchis felineus*	*Bithynia leachi*	Freshwater fish	Dog, cat, fox, chipmunk, beaver, Caspian seal, pig	Cholangitis, cirrhosis, cholangiocarcinoma	Germany, Greece, Poland, Romania, Italy, Spain, Belarus, Ukraine, Kazakhstan, Russia
*Metorchis conjunctus*	*Amnicola* sp.	Freshwater fish	Dog, cat, wolf, fox, coyote, raccoon, muskrat, mink, fisher	Fever, abdominal pain	Canada, Greenland
*Metorchis bilis*	*Bithynia* sp.	Freshwater fish	Wolf, white-tailed eagle, muskrat, otter, mink	Unknown	Central and Eastern Europe, Russia
*Metorchis orientalis*	*Parafossarulus* sp.	Cyprinoid fish	Dog, cat, duck, chicken, goose	Unknown	Japan, China, South Korea
*Amphimerus* sp.	Freshwater snail	Freshwater fish	Duck	Liver fibrosis, cirrhosis	Ecuador
*Amphimerus noverca*	Aquatic snail (?)	Freshwater fish	Dog, wolverine, pig	Periductal fibrosis	India
*Amphimerus pseudofelineus*	Aquatic snail (?)	Freshwater fish (?)	Dog, cat, coyote	Unknown	North and South Americas
*Pseudamphistomum truncatum*	Freshwater snail	Cyprinoid fish	Dog, cat, fox, seal, skunk, mink,otter	Liver pathology	Russia, Europe, North America
*Pseudamphistomum aethiopicum*	Freshwater snail	Fish	Unknown	Small tumour at intestinal wall	Ethiopia
*Dicrocoelium dendriticum*	Various snail species	Ant	Cattle, sheep, ox, deer, marmot, rabbit, goat	Mild cholangitis	Russia, Iran, Java, China, Lebanon, Syria, Egypt, Tunisia, France, Italy, Sweden, Germany, Switzerland, Spain, Hungary, Romania, Armenia, Czech Republic, Turkey, Uzbekistan, Iran, Saudi Arabia, Brazil, and USA
*Dicrocoelium hospes*	*Limicolaria* sp.	Ant	Cattle, sheep, zebus, goat, monkey	Probably mild cholangitis	DR Congo, Ghana, Sierra Leone, Nigeria
*Fasciola hepatica*	*Galba* sp.	Aquatic plant	Cattle, sheep, goat, European hare	Obstructive cholangitis, ectopic lesions	Ecuador, Bolivia, Chile, Peru, Cuba, Egypt, Portugal, France, Spain, Iran, Turkey, South Korea, Japan, China, Thailand, Vietnam
*Fasciola gigantica*	*Radix* sp.	Aquatic plant	Cattle, sheep, goat, buffalo, camel, pig, horse, domestic animal	Obstructive cholangitis, ectopic lesions	Japan, South Korea, China, Russia, Vietnam, Thailand, Malaysia, India, Iran, Sudan, Senegal, Chad, Ghana, Niger, Central African Republic, Tanzania, Kenya, USA (Hawaii)

a The geographical distributions of liver flukes are mostly referred from Chai and Jung (2019).

### Species involved

#### Clonorchis sinensis

*Clonorchis sinensis* (Cobbold, 1875) Looss, 1907 (the Chinese liver fluke) was first found in the bile passages of a Chinese carpenter in Calcutta, India, and an endemic focus was discovered in south China (Beaver *et al*., 1984). Eggs of this fluke were also found in the coprolites of human mummies (estimated date; 1647) in South Korea (Seo *et al*., 2007) and China (date back to the 5th century BCE) (Ye and Mitchell, 2016). This fluke infects the bile duct of humans and animals and can cause inflammation of the bile duct and gall bladder which leads to obstruction of the bile duct, cholangitis and cholecystitis (Chai and Jung, 2019). In severe cases, liver cirrhosis and cholangiocarcinoma may develop.

Phylogenetic relationships of small liver flukes have been studied using analysis of nuclear (18S rDNA, ITS region and 28S rDNA) and mitochondrial genes (*cox*1, *cox*2, *cox*3, *nd*1, ATPase subunit 6 and others) (Thaenkham *et al*., 2012; Besprozvannykh *et al*., 2018; Saijuntha *et al*., 2019, 2021; Duflot *et al*., 2021). Within the genus *Clonorchis,* there is only a single valid species, *C. sinensis*. ITS2 can clearly separate *C. sinensis, O. viverrini, O. felineus, Amphimerus* sp. and *Metorchis* spp. from the Heterophyidae and Cryptogonimidae flukes (Thaenkham *et al*., 2012; Besprozvannykh *et al*., 2018).

Freshwater snails take the role of the first intermediate host for *C. sinensis*, which includes *Parafossarulus manchouricus*, *P. anomalospiralis* and *Bithynia fuchsiana* (Chen *et al*., 1994). At least 113 species of freshwater fish, including *Pseudorasbora parva, Carassius* spp., *Cyprinus* spp., *Zacco* spp. and *Puntungia herzi*, are known to serve as the second intermediate hosts and the source of human and animal infections (Chen *et al*., 1994; Chai *et al*., 2005; Rim, 2005). Shrimps were also reported to be a second intermediate host (Chen *et al*., 1994).

The mode of infection is related to traditional food habits, in particular, the consumption of raw or improperly cooked freshwater fish (Chai *et al*., 2005). In South Korea, the major type of fish dish responsible for *C. sinensis* infection is the sliced raw freshwater fish with red pepper sauce (Chai *et al*., 2005). In southern China and Hong Kong, the major type of fish dish responsible is the morning congee (rice gruel) with slices of raw freshwater fish (Chen *et al*., 1994), whilst in the Guangdong Province of China, half-roasted or undercooked fish is commonly linked to infection (Chen *et al*., 1994).

The endemic areas are located mostly in the Far East and East Asia (Chen *et al*., 1994; Chai *et al*., 2005; Rim, 2005). In South Korea, a national survey in 2012 reported an egg positive rate of 1.9%, and the number of infected people was estimated to be about 1 million (Korea Association of Health Promotion, 2013). In China, a total of 24 major endemic localities were reported, with 12.5 million infected people nationwide (Chen *et al*., 1994; Hong and Fang, 2012; Lai *et al*., 2016). In Taiwan, the prevalence of clonorchiasis was high in some localities; however, the current status is unknown (Chai and Jung, 2019). In Vietnam, northern parts, especially along the Red River Delta, including Haiphong and Hanoi, are well-known endemic areas, with the number of infected people estimated at about 1 million (Rim, 1982a; Chai *et al*., 2005; Chai and Jung, 2019). In Russia, human infections were reported in the Amur River territory, although the exact prevalence is unknown; however, at least 1 million people are estimated to be infected (Hong and Fang, 2012). The total global number of infected people is estimated at about 20 million (Hong and Fang, 2012), and the number at risk is about 601 million (Garcia, 2016).

The metacercariae excyst in the duodenum, migrate through the ampulla of Vater to the distal biliary ducts, where they begin to lay eggs 3–4 weeks after infection (Beaver *et al*., 1984). Cholangitis and cholecystitis are the main pathological features in association with secondary bacterial infections (Rim, 1982a). The major histopathological features of the involved bile ducts include irregular bile duct dilatation, glandular hyperplasia, mucin-secreting cell metaplasia, cystic degeneration and periductal fibrosis (Rim, 1982*a*; 2005; Hong and Fang, 2012). In some patients, biliary cirrhosis and biliary obstruction with blockage of the common bile duct by adult worms or stones, or both, may occur (Beaver *et al*., 1984). The pathogenesis and pathology depend on the intensity of infection as well as the frequency of continuous reinfection over a period of years and the total length of the infection which can last for 15–20 years or longer (Beaver *et al*., 1984). The clinical symptoms include jaundice accompanied by transient urticaria and pain at the site of the liver; in heavy infections, weakness, lassitude, epigastric discomfort, paraesthesia, loss of weight, palpitation, tachycardia, diarrhoea and toxaemic symptoms may occur (Rim, 1982*a*). In chronic stages, suppurative cholangitis, biliary stone, pancreatitis, liver cirrhosis and cholangiocarcinoma are important complications (Rim, 1982a; Kim, 1984b).

*C. sinensis* is one of the well-known carcinogenic flukes that can cause cholangiocarcinoma of the bile duct (Rim, 1982*a*; 2005; Hong and Fang, 2012). The scientific evidence for this was first raised in Hong Kong, an endemic area of clonorchiasis, where it was shown that at least 15% of 200 primary cancers in the liver were induced by infection with *C. sinensis* (Hou, 1956). In South Korea, close correlations were also found between the cholangiocarcinoma incidence and the prevalence of *C. sinensis* infection in a southern endemic area (Rim, 1982a; Kim, 1984b).

In known endemic areas, it is relatively easy to diagnose *C. sinensis* infection through fecal examinations to detect parasite eggs. The daily number of eggs produced per worm was estimated at about 4000 (Rim, 1982*a*); therefore, the egg detectability in fecal examinations is considerably high. However, in areas previously unknown for FBT infections, the eggs should be differentiated from those of other small liver fluke species (such as *O. viverrini, O. felineus, Metorchis* spp., or *Amphimerus* spp.) and also of heterophyid (*Metagonimus yokogawai, Heterophyes nocens* and various others) and lecithodendriid-like trematode species (*Caprimorgorchis molenkampi* and others) (Lee *et al*., 1984; Chai and Lee, 2002; Chai, 2007). Serological tests, such as enzyme-linked immunosorbent assay (ELISA), and radiologic techniques, including ultrasound and CT, are also helpful for diagnosis (Hong and Fang, 2012). Detection of parasite DNA using varied genetic techniques is another method applicable for the diagnosis of clonorchiasis (Hong and Fang, 2012).

Treatment of *C. sinensis* infection can be done using a potent anthelmintic, praziquantel (Chai, 2013a), or alternatively, albendazole (Table 2). Although such treatment is effective at clearing the infection, fluke-induced bile duct pathology did not recover within 9–12 weeks after praziquantel treatment (Lee *et al*., 1988; Chai, 2013a). Control strategies include MDA of infected people in endemic areas, environmental sanitation (sterilization of feces and protection of fish ponds from contamination with night-soil), control of snail hosts, and health education to avoid eating raw or improperly cooked freshwater fish (Rim, 1982a).

**Table 2.** Drugs used for the treatment of FBT infections

Anthelmintic drug	Species of FBT	Drug regimen	Remark
Praziquantel	*Clonorchis sinensis*	25 mg kg^−1^ tid for 2 days	Individual treatment
		40 mg kg^−1^ single dose	mass treatment
	*Opisthorchis viverrini*	25 mg kg^−1^ tid for 1–2 days	Individual treatment
		40 mg kg^−1^ single dose	Mass treatment
	*Opisthorchis felineus*	25 mg kg^−1^ tid for 1 day	Individual treatment
		40 mg kg^−1^ single dose	Mass treatment
	*Dicrocoelium dendriticum*	20–25 mg kg^−1^ tid for 1–4 days	Satisfactory effects
	*Paragonimus* spp.	25 mg kg^−1^ tid for 2 days	Individual and mass treatment
	Intestinal flukes	10–20 mg kg^−1^ in single dose	Individual and mass treatment
Triclabendazole	*Dicrocoelium dendriticum*	10 mg kg^−1^ single dose	Satisfactory effects
	*Fasciola* spp.	10 mg kg^−1^ single dose	Repeated after 12–24 h in heavy infections
	*Paragonimus* spp.	10 mg kg^−1^ single dose	Repeated after 12–24 h in heavy infections
Niclosamide	*Fasciolopsis buski*	40 mg kg^−1^ daily for 1–2 days	Alcohol should be avoided
	*Heterophyes heterophyes*	Total 6 g over 3 alternate days	Repeated if necessary
	*Metagonimus yokogawai*	100 mg kg^−1^ daily for 1–2 days	Moderate efficacy
	*Haplorchis taichui*	40 mg kg^−1^ single dose	100% cure rate in experimental mice
Albendazole	*Clonorchis sinensis*	1200–1800 mg daily for 3–5 days	Cure rate; 84.6%
	*Opisthorchis viverrini*	800 mg daily for 3–7 days	Cure rare; 60–96%
	*Dicrooelium dendriticum*	400 mg bid for 7 days	Satisfactory effects
	*Metagonimus yokogawai*	400 mg daily for 2 days	Cure rate; 61.1%
	*Fasciola* spp.	1200 mg daily for 7 days	Triclabendazole-resistant cases

Information obtained from Kumchoo *et al*. (2007), Fürst *et al*. (2012*b*), Chai (2013*a*), Garcia (2016) and Chai *et al*. (2021*a*).

#### Opisthorchis viverrini *and* O. felineus

In the genus *Opisthorchis*, at least 53 nominal species were described (Nawa *et al*., 2013). Among them, 30 species were recorded from avian hosts, and the remaining 23 were from molluscs, fish, reptiles and mammals (Nawa *et al*., 2013).

*Opisthorchis viverrini* (Poirier, 1886) Stiles and Hassall, 1896 (the cat liver fluke) and *Opisthorchis felineus* (Rivolta, 1884) Blanchard, 1895 (the cat liver flukes) are known to infect humans (Rim, 1982b; Saijuntha *et al*., 2019). *O. viverrini* was first discovered in the liver of a civet cat (*Felis viverrini*) brought from India to France (Miyazaki, 1991). Its first human infection was reported by Leiper in 1911 from the autopsy of two prisoners in Chiang Mai, Thailand (Wykoff *et al*., 1965). *O. felineus* was first discovered in the liver of a cat (Miyazaki, 1991), and human infection was first reported by Winogradoff in 1892 in Tomsk, Siberia (Beaver *et al*., 1984). Eggs of *O. felineus* were found in the coprolites of humans and dogs in Russia (Slepchenko, 2020). Phylogenetic studies of *Opisthorchis* spp. have been performed using sequences of nuclear (18S rDNA, ITS region and 28S rDNA) and mitochondrial genes (*cox*1, *cox*2, *cox*3, *nd*1, ATPase subunit 6 and others) (Besprozvannykh *et al*., 2018; Saijuntha *et al*., 2019, 2021; Duflot *et al*., 2021). Molecular genetic investigations showed that *O. viverrini* is a species complex ‘*O. viverrini* sensu lato’, containing two evolutionary lineages with many cryptic species (morphologically similar but genetically distinct species) occurring in Thailand and Laos (Saijuntha *et al*., 2021).

These flukes can infect the bile duct of humans and animals and can cause hepatobiliary disorders, as in *C. sinensis* (Chai and Jung, 2019). *O. viverrini* seems to have a higher potential for inducing cholangiocarcinoma than *C. sinensis* (Chai *et al*., 2005; Chai and Jung, 2019). Regarding *O. felineus*, studies are required for a proper understanding of its carcinogenic potential (Maksimova *et al*., 2017).

The molluscan intermediate host of *O. viverrini* is freshwater snails, including *Bithynia goniomphalos, B. funiculata, B. siamensis* and *Melanoides tuberculata* (Chai and Jung, 2019). The second intermediate hosts are various species of freshwater fish, i.e. *Hampala dispar, Puntius orphoides*, *P. brevis*, *P. gonionotus, P. proctozysron* and *P. viehoever* (Rim, 1982b; Chai and Jung, 2019; Chai *et al*., 2019a). *O. viverrini* may survive for 10–20 years in the human host (Saijuntha *et al*., 2019).

With regard to *O. felineus*, the molluscan host is *Bithynia leachi* species complex (*B. leachi, B. troscheli* and *B. inflata*) distributed in Eastern Europe (Mordvinov *et al*., 2012). The second intermediate hosts are various species of freshwater fish, including the chub (*Idus melanotus*), tench (*Tinca tinca* and *T. vulgaris*), bream (*Abramis brama* and *A. sapa*), barbel (*Barbus barbus*) and carp (*Cyprinus carpio*) (Rim, 1982b).

The geographical distribution of *O. viverrini,* which is determined in close relationship with the distribution of the snail host, is mostly along the Mekong River basin and Indochina peninsula (Chai *et al*., 2005; Chai and Jung, 2019). In Thailand, *O. viverrini* is distributed mainly in the north and northeastern regions (Sithithaworn and Haswell-Elkins, 2003), however, the prevalence has been declining since the 1990s (Chai and Jung, 2019). By contrast, in Laos, mainly along the Mekong River, the prevalence has been reported to be high even after the 1990s, with the prevalence being 51–67% among the riparian villagers (Rim *et al*., 2003; Chai *et al*., 2007; Nakamura, 2017). In Vietnam, endemic areas are located in southern parts, especially along the Mekong River (Chai and Jung, 2019). In Cambodia, Phnom Penh Municipality, Takeo, Kratie, Stung Treng and Preah Vihear Provinces were reported to be low-grade endemic areas (Yong *et al*., 2014; Chai and Jung, 2019; Khieu *et al*., 2019). In Myanmar, a low prevalence of *O. viverrini* infection has recently been confirmed in a suburban population around Yangon (Sohn *et al*., 2019). The number of global people infected with *O. viverrini* is estimated at over 10 million (Sripa *et al*., 2018), with 80 million at risk for *Opisthorchis* spp. infections (Garcia, 2016).

*O. felineus* is known to be distributed from the Iberian Peninsula (Portugal and Spain) to Eastern Europe and West Siberia (Mordvinov *et al*., 2012). Many riverside areas, including the Ob, Irtysh, Ural and Volga Rivers, are the most important endemic localities; the highest prevalence of 20–60% or above was reported in areas of West Siberia (Fedorova *et al*., 2018). Human infections were recorded previously in Lithuania (before 1901), Poland (before 1937), Romania (before 1957) and Spain (before 1932) but recently no cases seem to occur in these countries (Mordvinov *et al*., 2012). However, in the last 50 years, human cases have been reported in some European Union (EU) countries, Eastern Europe, and Kazakhstan (Mordvinov *et al*., 2012; Pozio *et al*., 2013). In Italy, from 2003 to 2011, there were 8 small outbreaks of *O. felineus* infection involving a total of 211 people through consumption of raw fillet of the tench (*T. tinca*) fished from two lakes named Bolsena and Bracciano (Pozio *et al*., 2013). Thus, *O. felineus* infection is regarded as an emerging disease in Italy (Pozio *et al*., 2013). The worldwide estimated number of people infected with *O. felineus* is 1.2–1.6 million (Saijuntha *et al*., 2019).

The pathogenesis and pathology as well as clinical symptoms of *O. viverrini* and *O. felineus* infections are basically the same as those of *C. sinensis* infection. Cholangiocarcinoma due to *O. viverrini* infection accounts for about 15% of all liver cancers and is the second most frequent primary liver cancer worldwide (Saijuntha *et al*., 2019). The highest incidence of this disease occurs in northern Thailand, where 5000 cases of cholangiocarcinoma are diagnosed annually (Sripa *et al*., 2012; Saijuntha *et al*., 2019). In Laos and Vietnam, the precise incidence of cholangiocarcinoma due to *O. viverrini* infection has been unknown. However, the global ‘Cholangiocarcinoma Foundation’ recently launched a Vietnam Veterans project regarding developing cholangiocarcinoma among the veterans due to their military service in Vietnam in the 1970s (https://cholangiocarcinoma.org/vietnam-veterans/). In endemic areas of *O. felineus* infection in eastern Europe and Russia, around 400 cases of cholangiocarcinoma occur every year (Hotez and Alibek, 2011). No precise carcinogenic mechanisms of *Opisthorchis*-induced cholangiocarcinoma have been completely understood (Sripa *et al*., 2018). However, a two-stage carcinogenic process was proposed; some kinds of carcinogenic substances, including dimethylnitrosamine (DMN), as an ‘initiator’, and parasites, like *O. viverrini* and *O. felineus*, act as a ‘promoter’ (Flavell, 1981; Maksimova *et al*., 2017). This hypothesis has been further developed into a three-stage (multistage) theory, including initiation, promotion and tumour progression (Sripa *et al*., 2018).

The diagnosis of *O. viverrini* or *O. felineus* infection can be done by fecal examinations to detect eggs. The daily number of eggs produced per worm of *O. viverrini* was estimated at 160–900, considerably less than the figure of 4000 as reported for *C. sinensis* (Rim, 1982*a*, 1982*b*). Serological tests and fecal antigen detection using ELISA (Sripa *et al*., 2010; Teimoori *et al*., 2017) and radiologic techniques, including ultrasound (Pungpak *et al*., 1997; Kim *et al*., 2018) and CT (Mairiang and Mairiang, 2003), are helpful for the diagnosis of opisthorchiasis and related cholangiocarcinoma. Detection of parasite DNA is another method applicable for the diagnosis of opisthorchiasis (Lovis *et al*., 2009; Sripa *et al*., 2010).

Treatment of *O. viverrini* and *O. felineus* infection can be done using a potent anthelmintic, praziquantel (Chai, 2013a), or alternatively, albendazole (Table 2). For prevention and control, see *C. sinensis* section. It is noteworthy that the metacercariae of *O. felineus* are tolerant to low temperatures, drying and high salt concentration and can be killed only by a high temperature (Pakharukova and Mordvinov, 2016).

#### Metorchis *spp.*

*Metorchis conjunctus* (Cobbold, 1860) Looss, 1899 (the North American liver fluke) is a common parasite of carnivorous mammals, including sledge dogs (causing fatality), in northern Canada (MacLean *et al*., 1996). Asymptomatic sporadic human infections were reported in Canada since 1945, particularly in aboriginal populations from Quebec to Saskatchewan and the eastern coast of Greenland (Babbott *et al*., 1961; MacLean *et al*., 1996). In 1993, an outbreak of a common-source infection with this fluke, with acute clinical symptoms of fatigue, upper abdominal tenderness and pain, low-grade fever, headache, weight loss and anorexia, occurred among 19 Korean immigrants in a river north of Montreal, Canada; the patients consumed wild-caught fish (*Catostomus commersoni*) that had been undercooked (MacLean *et al*., 1996). Laboratory findings included high blood eosinophilia and raised liver enzymes (MacLean *et al*., 1996). However, serious liver diseases have not been reported in human infections.

*Metorchis bilis* (Braun, 1790) Odening, 1962 is a liver fluke of carnivorous mammals in Central and Eastern Europe and Western Siberia of Russia (Mordvinov *et al*., 2012). The geographical range of *M. bilis* considerably overlaps with that of *O. felineus* (Mordvinov *et al*., 2012). Human infections with *M. bilis* were first suggested by demonstrating serum antibodies to *M. bilis* in 51.3% of 37 patients (37.8% of them reacted against both *O. felineus* and *M. bilis* antigens) residing in the Novosibirsk area of Russia (Kuznetsova *et al*., 2000). Another serological study also demonstrated positive immunoreactivity of 29 (63.2%) of 46 small liver fluke egg-positive patients to both *O. felineus* and *M. bilis* antigens and of 3 (7.9%) patients only to *M. bilis* antigen (Fedorov *et al*., 2002). Now it seems that infections of both humans and piscivorous vertebrates by *M. bilis* are under-diagnosed (Sitko *et al*., 2016).

*Metorchis orientalis* Tanabe, 1920 is a small liver fluke of piscivorous birds and mammals in Japan, China and South Korea (Chai and Jung, 2019). The first report of human infection was published in Guangdong Province, China (Lin *et al*., 2001); 4 (4.2%) of 95 residents examined in Ping Yuan County were egg positive and from 2 purged patients a total of 12 adult flukes were recovered (Lin *et al*., 2001). However, the epidemiological status and potential impact of human infection are unknown (Chai and Jung, 2019). The geographical range of this fluke considerably overlaps with that of *C. sinensis* (Chai and Jung, 2019).

#### Amphimerus *spp.*

*Amphimerus* sp. (species undetermined) was found to be a human parasitic liver fluke in Ecuador. In 2009, 4 human fecal samples from indigenous Chachi communities along the Cayapas River on the northern coast of Ecuador were found to be positive for opisthorchiid eggs (Calvopiña *et al*., 2011). Next year, a follow-up survey was conducted in the same communities, and a high proportion, 71 (24%) of 297 fecal samples, of residents, were positive for the same eggs (Calvopiña *et al*., 2011). The biliary liquid was collected from 4 patients under duodenoscopy, and eggs identical to those found in the feces were found; they were treated with praziquantel followed by purging, and adult worms recovered were identified as a species of *Amphimerus* (Calvopiña *et al*., 2011). The patients had a history of consuming smoked or lightly cooked fish; the number of people living in endemic communities is about 24 000 (Calvopiña *et al*., 2011). A further study reported a very high prevalence of infection with this fluke in domestic cats and dogs living in Chachi communities, Ecuador (Calvopiña *et al*., 2015). Several species of freshwater fish were confirmed molecularly to have metacercariae of *Amphimerus* sp. (Romero-Alvarez *et al*., 2020). Human *Amphimerus* sp. infection is in most cases asymptomatic; however, it occasionally can cause non-specific generalized symptoms (Cevallos *et al*., 2017). Liver cirrhosis and pancreatitis occurred in cats and a cormorant infected with this fluke (Cevallos *et al*., 2017).

*Amphimerus noverca* (Braun, 1902) Baker, 1911 is a common parasite in the bile duct or pancreatic duct of dogs, wolverines and pigs in India (Beaver *et al*., 1984; Roy and Tandon, 1992). Human infection with *A. noverca* was mentioned two times (in 1876 and 1878) in India (Mas-Coma and Bargues, 1997). No further reports on human infections are available.

*Amphimerus pseudofelineus* (Rodriguez, Gomez Lince et Montalvan, 1949) Artigas and Perez, 1964 was originally reported under the name *Opisthorchis guayaquilensis* from 18 of 245 humans (based on eggs in feces) and several dogs (based on eggs in feces and adult worms) in a rural area of Ecuador by Rodriguez and colleagues (Mas-Coma and Bargues, 1997). Then, Thatcher (1970) transferred this fluke to the genus *Amphimerus*. This fluke is now known to be distributed in North and South America, probably taking aquatic snails shedding cercariae and fish harbouring encysted metacercariae (Mas-Coma and Bargues, 1997).

#### Pseudamphistomum truncatum *and* P. aethiopicum

*Pseudamphistomum truncatum* (Rudolphi, 1819) Lühe, 1908 is a species of liver or gall bladder fluke in mammals in Europe and North America (Yamaguti, 1971; Mas-Coma and Bargues, 1997). Its human infection was briefly mentioned in 1928 in Europe (Mas-Coma and Bargues, 1997). Later, in the Aleksee District of Tartaria, Russia, 31 human cases were diagnosed among 61 patients who had hepatobiliary system damage and had undergone bile analysis (Khamidullin *et al*., 1991). Human infections were also detected in five areas along the Volga, Kama and Belaya Rivers in Russia (Khamidullin *et al*., 1995). The eggs and metacercariae of *P. truncatum* can be morphologically differentiated from those of *O. felineus* (Mas-Coma and Bargues, 1997). The pathogenicity in humans is unknown but in otters and mink, *P. truncatum* can cause cholecystitis (Simpson *et al*., 2005; Sherrad-Smith *et al*., 2009).

*Pseudamphistomum aethiopicum* Pierantoni, 1942 was reported, on one occasion, to have caused a human infection with a small tumour in the intestinal wall (Yamaguti, 1971).

#### Dicrocoelium dendriticum *and* D. hospes

Within the genus *Dicrocoelium*, 5 species are currently recognized to be valid; *D. dendriticum* (Rudolphi, 1819) Looss, 1899, *D. chinensis* Tang and Tang, 1978, *D. hospes* Looss, 1907, *D. orientale* Sudarikov and Ryjikov, 1951 and *D. petrowi* Kassimov, 1952 (Manga-González and Ferreras, 2014).

*Dicrocoelium dendriticum* and *Dicrocoelium hospes* are known to cause human infections in rare instances. *D. dendriticum* (the lancet fluke) is a small liver fluke infecting domestic animals in the Northern coasts of Africa, Asia and the Americas (Mas-Coma and Bargues, 1997; Traversa *et al*., 2013; Manga-González and Ferreras, 2014). Using mitochondrial *nd*1 and *cox*1 sequences, *D. dendriticum* could be distinguished from *D. chinensis* and *D. hospes* (Hayashi *et al*., 2017; Khan *et al*., 2021). In human dicrocoeliasis, there may be true (genuine) and false (spurious) infections. The geographical localities where human infections were reported are very wide, and the incidence is undoubtedly underestimated (Mas-Coma and Bargues, 1997).

The first intermediate host includes more than 90 species of land snails, with *Cionella* (= *Cochlicopa*) *lubrica* as the most important species (Traversa *et al*., 2013). The cercariae are shed from the snails in clusters of thousands forming a so-called ‘slime ball’ (Traversa *et al*., 2013). These cercariae are ingested by ants (*Formica fusca, F. pratensis* and *F. rufibarbis*) (Traversa *et al*., 2013). Humans are infected accidentally by swallowing an infected ant together with food (Traversa *et al*., 2013).

In human infections, the pathogenicity depends on the number of flukes infected and the duration of infection, and in many instances, there may be no notable symptoms. However, a prolonged period of constipation or diarrhoea, nausea, vomiting, abdominal discomfort and epigastric pain may occur (Mas-Coma and Bargues, 1997). The diagnosis is based on the recovery of eggs in feces. However, an egg-positive result does not necessarily indicate a ‘true infection’, and a ‘spurious infection’ should be ruled out.

Serodiagnosis, including immunofluorescence, immunoblot, haemagglutination, complement fixation and ELISA have been introduced (Traversa *et al*., 2013; Dar *et al*., 2020). For treatment, albendazole (Magi *et al*., 2009), praziquantel (Drabick *et al*., 1988; Khandelwal *et al*., 2008), triclabendazole (Khandelwal *et al*., 2008; Ashrafi, 2010) and myrrh (herbal drug in Egypt; Mirazid®) (Abdul-Ghani *et al*., 2009) showed favourable results (Table 2).

*D. hospes* is a small liver fluke (more slender and smaller than *D. dendriticum*) infecting the bile duct and gall bladder of domestic animals in sub-Saharan Africa (Mas-Coma and Bargues, 1997). This liver fluke can also cause genuine and spurious infections in humans (Mas-Coma and Bargues, 1997). Two human patients in Sierra Leone (spurious infection was not ruled out) showed hepatitis-like symptoms; one patient had jaundice, and both had elevated bilirubin and transaminase levels (King, 1971). In animals, *D. hospes* does not seem to cause much damage even in heavy infections (Mas-Coma and Bargues, 1997). Diagnosis can be made by the recovery of eggs in feces; however, the eggs are morphologically indistinguishable from those of *D. dendriticum* and *Eurytrema pancreaticum* (Mas-Coma and Bargues, 1997). Anthelmintic drugs used against *D. dendriticum* may have similar effects on *D. hospes*.

#### Fasciola hepatica *and* F. gigantica

*Fasciola hepatica* Linnaeus, 1758 (the sheep liver fluke) infects the bile duct of domestic animals, including cattle and sheep, and occasionally humans (Beaver *et al*., 1984; Mas-Coma *et al*., 2005; Liu and Zhu, 2013). The parasite life cycle, biology, pathogenesis, pathology, epidemiology and clinical symptoms are similar between *F. hepatica* and *F. gigantica* (Chai and Jung, 2019). Hybrid forms between *F. hepatica* and *F. gigantica* have been found in Asia (Tang *et al*., 2021). Humans are accidental hosts (Liu and Zhu, 2013). They are infected through eating aquatic vegetables or raw liver of infected livestock animals (Liu and Zhu, 2013). The history of human infection with *Fasciola* spp. seems to be very long, since archaeological studies in ancient mummies in Egypt revealed remains of parasites (possibly part of *F. hepatica* worm) in liver tissue (David, 1997). Sporadic cases of human *Fasciola* spp. infections (genuine or spurious infection unclear) were reported in Egypt in the 1950s (Kuntz *et al*., 1958), and an epidemic occurred in France in 1956 (Mas-Coma *et al*., 2005). An extensive review of human fascioliasis was performed by Chen and Mott (1990) in which 2595 cases from over 40 countries in Europe, Asia, Western Pacific, the Americas and Africa from 1970 to 1990 were analysed. Now human fascioliasis (*F. hepatica* and *F. gigantica*) is recognized by the WHO as an important FBT disease with an estimated 2.4 million people infected annually and 180 million at risk in over 61 countries (Haseeb *et al*., 2002). Large-scale epidemics occurred in France, Egypt and Iran (Parkinson *et al*., 2011).

In the genus *Fasciola*, three species are currently recognized; *F. hepatica*, *F. gigantica* Cobbold, 1855, and *F. nyanzae* Leiper, 1910 (Mas-Coma *et al*., 2009; Rajapakse *et al*., 2020). Among them, the most common species worldwide are *F. hepatica, F. gigantica* and their hybrid forms (Mas-Coma *et al*., 2009, 2019). In Europe, the Americas and Oceania, only *F. hepatica* is found, whereas both species and their hybrids are commonly found in Asia and Africa (Mas-Coma *et al*., 2005, 2019). For phylogenetic analyses of *Fasciola* spp. worms, sequences of nuclear rDNA, including ITS1-5.8S-ITS2 and 28S rDNA, and mitochondrial genes, including *nd*1 and *cox*1, have been found to be useful and popularly used to differentiate *F. hepatica, F. gigantica* and the intermediate forms (Mas-Coma *et al*., 2009, 2018, 2019). Molecular analyses of five Korean *Fasciola* worms from cattle using ITS-2 and 28S rDNA revealed that one possessed the *F. hepatica*-type sequence, two *F. gigantica*-type sequences, and two possessed sequences of both types (Agatsuma *et al*., 2000). An adult specimen recovered from a Korean male patient revealed *F. hepatica* ITS-1 genotype (Kang *et al*., 2014). In China, using ITS-2 sequences to perform RFLP analysis, the *Fasciola* worms from Sichuan Province represented *F. hepatica*, those from Guangxi Province represented *F. gigantica*, and the ones (from sheep) from Heilongjiang Province may represent an ‘intermediate genotype’ (Huang *et al*., 2004). In Japan, both ITS-1 and ITS-2 were useful to discriminate Fsp1 (*F. hepatica*), Fsp2 (*F. gigantica*) and Fsp1/2 (intermediate form), but *nd*1 and *cox*1 could not discriminate the intermediate form properly (Itagaki *et al*., 2005). Also in Europe, ITS-2 showed low variability for both fasciolid species and appeared to furnish valuable information (Mas-Coma *et al*., 2009). It is also notable that in Asian countries there are aspermic *Fasciola* flukes that may reproduce parthenogenetically (Itagaki *et al*., 2005).

A wide variety of freshwater snails that belong to the Lymnaeidae play the role of the first intermediate host (Bargues and Mas-Coma, 2005). In Asia, Europe, Africa and the Western Pacific, *Galba truncatula* (syn. *Lymnaea truncatula*) and *Austropeplea ollula* (syn. *Austropeplea viridis*) snails are most frequently involved (Bargues and Mas-Coma, 2005; Chai and Jung, 2019). Encysted metacercariae of *F. hepatica* are found on leaves of aquatic vegetables (watercress, alfalfa, water lettuce etc.) or green vegetation, bark or other smooth surfaces above or below the waterline (Beaver *et al*., 1984; Mas-Coma and Bargues, 1997; Liu and Zhu, 2013). People in Latin America, France and Algeria frequently acquire this disease by eating raw watercress (*Nasturtium officinale*) (Beaver *et al*., 1984). In Asia, water bamboo, water caltrop and morning glory were suspected to be important sources of human infections. Recently in South Korea, the water parsley (water dropwort) and its juice have been suspected as the potential source of sporadic human infections (unpublished data). Humans can also be infected by eating raw livers of infected animals containing immature or young worms, and these worms attach to the pharyngeal mucosa and cause pain, bleeding, oedema and dyspnoea, and this condition was once called ‘halzoun’ (Beaver *et al*., 1984).

*F. hepatica* is distributed almost worldwide, especially where extensive sheep and cattle raising is popularly done (Liu and Zhu, 2013). Human fascioliasis is one of the major public health problems in northern Africa, western Europe, Andean countries, the Caribbean area and the Caspian areas (Mas-Coma and Bargues, 1997). In South Korea, Japan, China, Thailand and Vietnam, sporadic cases have been reported (Chen and Mott, 1990; Mas-Coma and Bargues, 1997; Chai and Jung, 2019). It is of particular note that the highest prevalence (up to 72% by coprological and 100% by serological tests) and intensity ever have been found in some communities in the Northern Bolivian Altiplano (Mas-Coma and Bargues, 1997).

The pathological changes in the liver and bile duct of infected humans or animals depend primarily on the number of flukes infected (Chen and Mott, 1990), and the pathology in human infections is similar to that reported in animals (Mas-Coma and Bargues, 1997). However, in about 50% of cases, human fascioliasis is asymptomatic probably due to infection with a low number of flukes (Liu and Zhu, 2013). In rare instances (<10% of all patients), ectopic fascioliasis can occur (Beaver *et al*., 1984; Mas-Coma and Bargues, 1997). The pathogenicity of *F. hepatica* and *F. gigantica* in humans is not recognizably different, although in sheep *F. gigantica* was found to be more pathogenic (Mas-Coma and Bargues, 1997).

After excystation in the duodenum or jejunum of their definitive host, the metacercariae of *F. hepatica* migrate through the intestinal wall and peritoneal cavity, and then penetrate into the liver parenchyma through Glisson's capsule and finally reside in the bile duct and gall bladder or go astray to other ectopic locations (Beaver *et al*., 1984; Liu and Zhu, 2013). They can cause mechanical damage with focal haemorrhage, inflammatory reactions and necrotic lesions (Beaver *et al*., 1984). Adult worms may live between 9 and 13.5 years in humans (Mas-Coma *et al*., 2005). Stone formation in the bile duct or gall bladder is frequent, but liver cirrhosis is less common (Mas-Coma *et al*., 2019). The pathogenesis of ectopic fascioliasis is not fully understood. The ectopic locations include the skin, subcutaneous tissue, skeletal muscle, blood vessel, lungs, orbit, ventricles of the brain, stomach, appendix, pancreas, intestinal wall, heart, spleen, epididymis and lymph nodes (Beaver *et al*., 1984; Chen and Mott, 1990).

The diagnosis of human fascioliasis can be done directly using parasitological techniques and indirectly by immunological tests and radiologic images, including ultrasound, CT, MRI and radioisotope scanning (Mas-Coma *et al*., 2019).

Triclabendazole is currently the drug of choice for human fascioliasis (Fairweather, 2009; Mas-Coma *et al*., 2019) (Table 2). A problem related to triclabendazole is the appearance of drug resistance particularly in livestock animals; it was first described in Australia and then in many European and South American countries (Mas-Coma *et al*., 2019). Praziquantel was also used for human fascioliasis but treatment failure was experienced even at high doses (Mas-Coma *et al*., 2019). Albendazole, nitazoxanide, Mirazid® and artesunate are alternative drugs for potential use in human and animal fascioliasis (Mas-Coma *et al*., 2019; Chai *et al*., 2021a). Infection may be prevented by strict avoidance of consuming watercress and other metacercaria-carrying aquatic plants in endemic areas (Mas-Coma *et al*., 2019).

*Fasciola gigantica* Cobbold, 1855 (the giant cattle liver fluke) is a large liver fluke species infecting the bile duct of domestic animals, including cattle and sheep, and occasionally humans (Beaver *et al*., 1984; Mas-Coma and Bargues, 1997; Liu and Zhu, 2013). In Asia and Africa, *F. hepatica, F. gigantica* and their hybrid forms are found, but in Europe, the Americas and Oceania, only *F. hepatica* is found (Mas-Coma *et al*., 2005, 2019). The parasite's life cycle, biology, pathogenesis, pathology, epidemiology and clinical symptoms are similar to those of *F. hepatica* (Chai and Jung, 2019). The most important snail host for *F. gigantica* is *Radix auricularia* (Liu and Zhu, 2013). The absence of *F. gigantica* in the New World may be explained by the absence of these snail species (*Radix* spp.) (Mas-Coma *et al*., 2009). The metacercariae are encysted on the leaves of aquatic plants (Garcia, 2016).

## Lung flukes

Paragonimiasis is a zoonotic disease caused by lung flukes of the genus *Paragonimus* (Chai and Jung, 2018). As of 1999, more than 50 nominal species had been described in this genus (Blair *et al*., 1999a; Narain *et al*., 2010; Doanh *et al*., 2013a). However, 16–17 of them were synonymized with the others, and the remaining 36 species were regarded as valid or potentially valid (Blair *et al*., 1999a). Thereafter, five new species were described from Asia and Africa; *P. vietnamensis* (Doanh *et al*., 2007), *P. pseudoheterotremus* (Waikagul, 2007), *P. sheni* (Shan *et al*., 2009), *P. gondwanensis* (Bayssade-Dufour *et al*., 2014) and *P. kerberti* (Bayssade-Dufour *et al*., 2015). Among the 41 nominal species (or subspecies), at least 9 are known to cause human infections, including *P. westermani, P. africanus, P. gondawanensis, P. heterotremus, P. kellicotti, P. mexicanus, P. skrjabini, P. skrjabini miyazakii* and *P. uterobilateralis* (Table 3) (Chai, 2013*b*; Bayssade-Dufour *et al*., 2014; Chai and Jung, 2018; Blair, 2019). The lung flukes can cause pulmonary as well as extrapulmonary infections in humans. The global number of people infected with *Paragonimus* spp. was estimated at about 23 million in 48 countries (mostly in China) with 292 million people at risk worldwide (Blair, 2019).

**Table 3.** Lung flukes infecting humans with biological, clinical characteristics and geographical distribution

Species of flukes	First intermediate host (snail)	Second intermediate host	Reservoir host	Major clinical characteristics	Geographical distribution^a^
*Paragonimus westermani*	*Semisulcospira* spp. *Juga* spp. *Melanoides tuberculata* *Brotia* spp. *Tarebia granifera*	Crab, crayfish	Dog, cat, pig, leopard, tiger, fox, wolf, opossum, mink, monkey	Haemoptysis, bronchiectasis	China, Taiwan, South Korea, Japan, Southeast Siberia (Russia), The Philippines, Malaysia, Thailand, Cambodia, Laos, Vietnam, Myanmar, Sri Lanka, India, Nepal, Pakistan, Papua New Guinea
*Paragonimus africanus*	*Potadoma freethii Melania* sp.	Crab	Dog, mongoose, civet, drill, monkey	Haemoptysis, bronchiectasis	Guinea, Cameroon, Nigeria, Ivory Coast
*Paragonimus gondwanensis*	Unknown	Crab	Cat, civet	Unknown	Cameroon
*Paragonimus heterotremus*	Various snail species	Crab	Dog, cat, monkey, Mongolian gerbil, rabbit	Haemoptysis, bronchiectasis	China, Vietnam, Laos, Thailand, Myanmar, Sri Lanka, India
*Paragonimus kellicotti*	*Pomatiopsis* spp.	Crab, crayfish	Dog, cat, pig, skunk, red fox, coyote, mink, bobcat	Haemoptysis, bronchiectasis	Canada, USA
*Paragonimus mexicanus*	*Aroapygrus* spp.	Crab	Dog, cat, opossum	Haemoptysis, bronchiectasis	Mexico, Peru, Ecuador, Coasta Rica, Panama, Guatemala
*Paragonimus skrjabini*	Various snail species	Crab	Dog, cat, rat, mouse, weasel, monkey	Ectopic lesions	China, Vietnam, northeastern India
*Paragonimus skrjabini* *miyazakii*	*Oncomelania* sp.	Crab	Dog, cat, marten, weasel, wild boar	Haemoptysis, ectopic lesions	Japan
*Paragonimus uterobilateralis*	Unknown	Crab	Dog, cat, otter, mongoose, swamp, shrew	Haemoptysis, bronchiectasis	Cameroon, Nigeria, Liberia, Guinea, Ivory Coast, Gabon

a The geographical distributions of lung flukes are mostly referred from Chai and Jung (2019).

### Species involved

#### Paragonimus westermani

*Paragonimus westermani* (Kerbert, 1878) Braun, 1899 (the Oriental lung fluke) was originally reported from the lungs of a Bengal tiger (India) that died in the Zoological Garden in Amsterdam (Beaver *et al*., 1984). Since then, this species has been reported mainly in Asian countries (Blair *et al*., 1999a; Narain *et al*., 2010). Human infections were first found in a Portuguese residing in Taiwan through recovery of adult worm (s) after autopsy in 1880 and also in Japanese patients through detecting eggs from bloody sputum in 1880 and then adult worms in 1883 in Japan (Beaver *et al*., 1984). Now human infections with this lung fluke continue to occur in Asian countries, including China, Taiwan, Japan, South Korea, Far Southeast Russia, the Philippines and recently India (Chai, 2013*b*; Singh *et al*., 2015; Blair, 2019). While human infections may be decreasing in Japan and South Korea, new endemic foci have been detected in several other countries (Yoshida *et al*., 2019). Morphologically *P. westermani* differs from other *Paragonimus* species in the patterns of lobation of the ovary and testes (Miyazaki, 1991). The ovary of *P. westermani* has 6 simple lobes, whereas that of *P. mexicanus* or *P. ohirai* displays many delicate branches (Miyazaki, 1991).

*Paragonimus siamensis* was described as a new species from experimental cats infected with metacercariae from the freshwater crab *Parathelphusa germaini* in Thailand (Miyazaki and Wykoff, 1965). Later, this lung fluke was found in animals in Sri Lanka (Kannagara and Karunaratne, 1969), India (Devi *et al*., 2013) and the Philippines (Yoshida *et al*., 2019). An adult specimen recovered from a New Guinea native man in 1926 was preserved in School of Public Health and Tropical Medicine, Australia, and it was restudied and assigned as *P. siamensis* (Wang *et al*., 2011). *Filopaludina martensi martensi* snails are the first intermediate host (Yaemput *et al*., 1994), and freshwater crabs, including *Ceylonthelphusa rugosa,* play the role of the second intermediate hosts (Blair *et al*., 1999a). Recent molecular studies reported that *P. siamensis* is nested within the *P. westermani* complex (Devi *et al*., 2013; Blair, 2019).

Phylogenetic studies revealed that *P. westermani* is a species complex comprising of two groups; the East Asia group (Japan, South Korea, China and Taiwan) and the Southeast Asia group (Malaysia and the Philippines) based on morphological and molecular data (Blair *et al*., 1997; Doanh *et al*., 2009). However, recent discoveries of *P. westermani* in Thailand (Sugiyama *et al*., 2007), India (Tandon *et al*., 2007) and Sri Lanka (Iwagami *et al*., 2008) provided evidence that *P. westermani* complex was constructed with more than two groups (Blair *et al*., 2007).

The first intermediate host is variable species of freshwater snails (Table 3). The second intermediate host is crustaceans, including freshwater crayfish *Cambaroides* spp. (*C. similis, C. dauricus* and *C. schrenki*), *Eriocheir* spp. crabs (*E. japonicus* and *E. sinensis*) and a variety of other crab species belonging to the family Potamidae or Parathelphusidae (Blair *et al*., 1999a). In China, Taiwan and South Korea, freshwater shrimps (*Macrobrachium nipponensis* and other species) were also reported as the second intermediate hosts (Blair *et al*., 1999a). There are several kinds of paratenic hosts, for example, wild boars, bears, wild pigs and rats; in these hosts worms do not mature to be adults but remain at a juvenile stage; they can be an important source of human infection (Miyazaki and Habe, 1976; Shibahara *et al*., 1992). The egg-laying capacity of *Paragonimus* worms was reported to be 11 000– 104 000 eggs per day per worm (EPD/worm) in experimental dogs (Yokogawa, 1965).

The principal mode of human *P. westermani* infection is consumption of raw or improperly cooked (pickled) freshwater crabs or crayfish (Chai, 2013*b*). In Asian countries, famous dishes causing human paragonimiasis have been known, for example, ‘drunken crab’ in China, ‘Kejang (= sauced crab)’ in South Korea, ‘Oboro-kiro (= crab juice soup)’ in Japan, ‘Goong ten (= raw crayfish salad)’ in Thailand, and ‘Kinuolao (= raw crab)’ in the Philippines (Kim, 1984a; Nakamura-Uchiyama *et al*., 2002). It is of note that freshwater crab or crayfish juice was used in South Korea and Japan for traditional treatment of febrile diseases, such as measles, asthma and urticaria (Kim, 1984a; Nakamura-Uchiyama *et al*., 2002). This kind of practice was formerly an important mode of contracting paragonimiasis, especially in children. Another important mode of infection in Japan is ingestion of raw or undercooked boar meat containing *P. westermani* metacercariae or juvenile worms (Miyazaki and Habe, 1976; Shibahara *et al*., 1992; Nakamura-Uchiyama *et al*., 2002). In paratenic hosts, worms do not mature and stay in muscles and tissues; when they were eaten by humans, they could develop into adult worms (Nakamura-Uchiyama *et al*., 2002; Chai, 2013*b*).

The geographical distribution of *P. westermani* is wide, including East Asia, Southeast Asia, South Asia (including India) and even the Western Pacific (Blair, 2019). Human cases of *P. westermani* were reported also in the USA (Fried and Abruzzi, 2010; Boland *et al*., 2011); however, the existence of the life cycle of *P. westermani* in North America needs to be verified.

When the definitive host, including humans, consumes crab or crayfish meat containing metacercariae, they excyst in the duodenum and penetrate into the intestinal wall; during this process they mature into juvenile flukes (Chai and Jung, 2018). The juvenile flukes enter the inner wall (abdominal muscle) of the abdominal cavity and reappear in the abdominal cavity; they then penetrate the diaphragm and enter the pleural cavity in about 14 days after infection (Yokogawa, 1965). In the pleural cavity, two worms mate and then move to the lung parenchyma where a fibrous cyst called the ‘worm cyst’ or ‘worm capsule’ develops around them (Narain *et al*., 2010; Chai, 2013*b*). The two worms exchange sperm and produce eggs within the worm cyst, which accumulate for some time in acute stages but in chronic stages when tissue necrosis occurs around the cyst, the eggs escape from the cyst into small bronchioles (Yokogawa, 1965; Blair *et al*., 1999a). There are triploid and tetraploid forms of *P. westermani* which do not produce sperm and reproduce parthenogenetically (Miyazaki, 1991; Terasaki *et al*., 1995; Agatsuma *et al*., 2003). The patients may undergo variable clinical types, including pulmonary, thoracic, abdominal, cerebral, spinal and cutaneous paragonimiasis (Nakamura-Uchiyama *et al*., 2002; Chai, 2013*b*).

In pulmonary infections, *P. westermani* worms lie in worm cysts of host origin, 1–2 cm in diameter, within the lung parenchyma (Blair *et al*., 1999a). The lung lesions can be classified into infiltrative, nodular and cavitating shadow types, or combination of these types (Nakamura-Uchiyama *et al*., 2002). In early stages of infection, there is no exit from the worm cyst, and eggs as well as excretions, metabolic products and tissue debris may gather progressively within the cyst, which becomes distended, and the cyst wall becomes thick and fibrotic (Yokogawa, 1965). In chronic stages, eggs can be demonstrated in the blood-tinged portion of the sputum (Chai, 2013*b*). The earliest possible clinical manifestations by day 15 after infection include abdominal pain, fever, chill, fatigue and diarrhoea (Choi, 1990; Nakamura-Uchiyama *et al*., 2002; Procop, 2009). Eosinophilia of up to 25% can occur at 2 months after infection (Choi, 1990; Procop, 2009). In chronic stages, the most common and important manifestations include chronic cough, rusty-coloured sputum (= haemoptysis), chest pain, dyspnoea and crepitation (Im *et al*., 1993; Nakamura-Uchiyama *et al*., 2002; Procop, 2009). In chest radiographs, cavitating lesions called ‘ring shadows’ or ‘cysts’ are commonly seen (Im *et al*., 1993). Pleural effusion is frequently seen in Korean and Japanese patients (Im *et al*., 1993; Nakamura-Uchiyama *et al*., 2002).

The extrapulmonary migration of *Paragonimus* worms has been suggested to occur due to several reasons. The most important reason is the complex migration route of the worms (Chai, 2013*b*). Another reason was suggested to be seeking sexual partners to exchange sperm (Miyazaki, 1991). The brain is the most commonly involved ectopic site, although the mechanisms and route of worm migration to the brain are not well understood (Choi, 1990).

Cerebral paragonimiasis occurs quite commonly in *P. westermani* infection, about 1% of all paragonimiasis patients (Oh, 1969). Five major symptoms include the Jacksonian type seizure, headache, visual disturbance, motor and sensory disturbances, and 5 major signs are optic atrophy, mental deterioration, hemiplegia, hemi-hypalgesia and homonymous hemianopsia (Oh, 1969). Intracranial haemorrhage can also occur though rare in the incidence (Choo *et al*., 2003; Koh *et al*., 2012). In skull radiography, cerebral calcification is the most commonly encountered finding, and temporal, occipital and parietal lobes of the brain are the predilection sites (Oh, 1968b). Compared with cerebral infections, spinal paragonimiasis is relatively rare (Oh, 1968a). The predilection site is extradural areas of the thoracic level (Choi, 1990).

Abdominal paragonimiasis is probably more common than cerebral and spinal infections (Meyers and Neafie, 1976). The affected organs include the abdominal wall (muscle), peritoneal cavity, liver, spleen, pancreas, heart, greater omentum, appendix, ovary, uterus, scrotum, inguinal regions, thigh and urinary tract (Chai, 2013*b*). Cutaneous and subcutaneous paragonimiasis are very rare compared to pleuropulmonary and other ectopic types (Chai, 2013*b*). *P. westermani* can cause abscesses and ulcers in the skin or subcutaneous tissue, but *P. skrjabini* can cause migrating subcutaneous nodules (Meyers and Neafie, 1976).

Conventional methods to diagnose human *Paragonimus* infections are microscopic examinations of the sputum or fecal samples for detecting eggs, chest radiography to observe the lung lesions (differential diagnosis needed with tuberculosis and lung cancers), and serological tests, including intradermal test, indirect haemagglutination test, ELISA, and others to detect antibodies (Lee *et al*., 2003; Sugiyama *et al*., 2013; Chai, 2013*b*). Sputum eggs can usually be detected in chronic cases when the worm cyst is ruptured and connected to bronchioles. In children, aged-people or handicapped individuals, sputum is frequently swallowed, and in such cases, eggs could be detected in feces (Chai, 2013*b*). Among the serological tests, ELISA (such as microplate ELISA and multiple-dot ELISA) is so far the most reliable tool because this assay method shows high sensitivity and high specificity (Lee *et al*., 2003; Yoshida *et al*., 2019). Molecular methods, including PCR and DNA sequencing, have become useful for specific identification of *Paragonimus* eggs and worms (Sugiyama *et al*., 2013). Other methods, including PCR-RFLP, multiplex PCR, random amplified polymorphic DNA (RAPD), and DNA hybridization, have also been applied for specific identification of *Paragonimus* (Narain *et al*., 2010; Sugiyama *et al*., 2013).

Praziquantel is the drug of choice for treating paragonimiasis (*P. westermani* and other *Paragonimus* spp.), including cerebral infections (Chai, 2013*b*) (Table 2). Rarely, allergic reactions may occur following praziquantel treatment; however, such patients can be successfully treated by desensitization to praziquantel (Kyung *et al*., 2011). Triclabendazole is a new promising drug for the treatment of pulmonary paragonimiasis (Keiser *et al*., 2005). However, its efficacy on cerebrospinal paragonimiasis remains to be determined (Chai, 2013*b*). Prevention of contamination of the crab and crayfish with the cercariae of *P. westermani* depends on environmental control of surface waters through the elimination of freshwater snails. Long-term health education of young people to avoid eating raw or improperly cooked crayfish or crabs seems to be one of the best ways to prevent *P. westermani* infection in endemic areas.

#### Paragonimus africanus

*Paragonimus africanus* Voelker and Vogel, 1965 was originally described from the mongoose in Cameroon and is now known to be distributed throughout sub-Saharan Africa (Narain *et al*., 2010). Up to 2008, it was estimated that there had been 2295 confirmed cases of human paragonimiasis in Africa mostly due to *P. africanus* and *P. uterobilateralis* with the vast majority occurring in Nigeria and Cameroon (Aka *et al*., 2008; Cumberlidge *et al*., 2018). Freshwater crabs *Liberonautes* spp. and *Sudanonautes* spp. carry the metacercariae (Aka *et al*., 2008). Its morphological characters include the distinctly larger oral sucker than the ventral sucker, a delicately branched ovary, and highly branched testes which are significantly larger than the ovary (Narain *et al*., 2010). Uncooked crab meat is an important source of human infection (Aka *et al*., 2008). Nucleotide sequences of ITS2 and *cox*1 have been used for specific diagnosis (as *P. africanus*) of fecal eggs from monkeys (Friant *et al*., 2015) and humans (Nkouawa *et al*., 2009). Clinical manifestations are similar to *P. westermani* infection. Cerebral infection has been suspected but never proved (Aka *et al*., 2008). Sputum and stool examinations to detect eggs are the main diagnostic procedures.

#### Paragonimus gondwanensis

*Paragonimus gondwanensis* Bayssade-Dufour *et al*., 2014 was described as a new species from humans (only eggs) and carnivorous mammals (adult worms) in Cameroon (Bayssade-Dufour *et al*., 2014). This species is morphologically distinct from all other *Paragonimus* species in having a very short excretory bladder, once used as a character for a different genus, *Euparagonimus* (Bayssade-Dufour *et al*., 2014). However, molecular data are lacking, and the validity of this species remains to be determined (Rabone *et al*., 2021). The second intermediate host is freshwater crabs, *Sudanonautes africanus* (Bayssade-Dufour *et al*., 2014).

#### Paragonimus heterotremus

*Paragonimus heterotremus* Chen and Hsia, 1964 was first discovered from rats in China and is now known to be distributed in China, Indochina peninsula, Sri Lanka and India (Sing *et al*., 2009; Narain *et al*., 2010; Yoshida *et al*., 2019). In 2007, *P. pseudoheterotremus* was reported as a new species from a cat experimentally infected with the metacercariae in crabs from a mountainous area of Thailand (Waikagul, 1997). Later, however, this species is considered a geographical variation of the *P. heterotremus* complex, based on molecular studies using ITS2 and *cox*1 sequences (Sanpool *et al*., 2013; Doanh *et al*., 2015; Tantrawatpan *et al*., 2021). Human infection with *P. heterotremus* was first identified by the recovery of an adult worm from a 13-year-old boy in Nakorn-Nayok Province, Thailand in 1965 (Miyazaki and Harinasuta, 1966). In the same year, eggs presumed to be of *P. heterotremus* were detected in the bloody sputum of a patient in Guangxi, China (Zhou *et al*., 2021). Since then, a lot of human cases have been reported (Miyazaki, 1991; Singh *et al*., 2009; Doanh *et al*., 2013a). Freshwater snails, *Assiminea* sp., *Oncomelania hupensis* and *Neotricula aperta* (syn. *Tricula aperta*), are known to serve as the first intermediate host, and freshwater crabs, including *Larnaudia beusekomae, Siamthelphusa paviei* and *Potamiscus smithianus* (Thailand), *Potamon flexum* and *Sinolapotamon patellifer* (China), take the role of the second intermediate hosts (Blair *et al*., 1999a). In adult specimens, the ventral sucker is characteristically small, about a half the diameter of its oral sucker (Narain *et al*., 2010). The ovary and testes are both delicately branched (Miyazaki, 1991). The pathology and clinical manifestations in *P. heterotremus* infection is similar to those seen in *P. westermani* infection. The diagnosis is based on the recovery of eggs in bloody sputum, serology, chest radiography and molecular genetic analysis.

#### Paragonimus kellicotti

*Paragonimus kellicotti* Ward, 1908 was originally discovered in a cat and a dog in the USA (Blair *et al*., 1999a; Procop, 2009). The lobation of the ovary and testes is more complex than in *P. westermani* (Procop, 2009). Now this species is known to occur in central and eastern parts of the USA and adjacent areas of Canada (Procop, 2009). Beaver *et al*. (1984) considered the first human case to be a German labourer who worked in the USA in the 1890s and had eaten crayfish. Another case was a Canadian man aged 51 years who had never been outside of Quebec and complained of systemic and pulmonary symptoms (Béland *et al*., 1969; Coogle *et al*., 2021). Thereafter, from 1984 until 2017, a total of 20 human cases have been documented (Lane *et al*., 2009, 2012; Procop, 2009; Fried and Abruzzi, 2010; Coogle *et al*., 2021). Molecular studies were done on ITS2, *cox*1 and other genetic loci (Blair *et al*., 1999b; Fischer *et al*., 2011; McNulty *et al*., 2014). Based on ITS2 and *cox*1 sequences, *P. kellicotti* was genetically closest to *P. macrorchis* and *P. mexicanus*, and then to *P. heterotremus*, but distant from *P. westermani* and *P. siamensis* (Blair *et al*., 1999b). The draft genome of *P. kellicotti* has been established and analysed in comparison with those of *P. westermani, P. skrjabini miyazakii* and *P. heterotremus* (Rosa *et al*., 2020). The second intermediate host is the crayfish of *Cambarus bartoni, C. robustus* and *C. virilis*, *Orconectes propinquus, O. rusticus* and *Procambarus blandingi acutus*, or crabs *Geothelphusa dehaani* (Blair *et al*., 1999a). In the USA, frozen or pickled crabs available at markets are suspected to be the source of *P. kellicotti* infection (Procop, 2009). Pleuropulmonary infection is dominant in *P. kellicotti* infection, and the most frequent clinical manifestations among 21 North American patients were cough, pleural effusion, fever, fatigue or malaise, weight loss, chest pain, dyspnoea, haemoptysis and eosinophilia (Coogle *et al*., 2021). Their diagnosis was based on microscopic examinations of sputum, pleural fluid or bronchoalveolar lavage and serological tests using complement fixation test, immunoblot or western blot (Coogle *et al*., 2021).

#### Paragonimus mexicanus

*Paragonimus mexicanus* Miyazaki and Ishii, 1968 (syn. *Paragonimus peruvianus*) was first described from opossums in Colima, Mexico (Miyazaki and Ishii, 1968). Its characteristic morphology includes a somewhat larger oral sucker than the ventral sucker and a strongly lobed ovary and two testes (Miyazaki, 1991). Metacercariae have no cyst wall (Sugiyama *et al*., 2013). *P. mexicanus* is now known to be distributed in Central and South Americas (Blair *et al*., 1999a). A human case reported by Báez M. and Galán J. in 1961, a 35-year-old Mexican man from whom eggs were detected in lung tissue, was believed to have been due to *P. mexicanus* infection (Miyazaki and Ishii, 1968). In Costa Rica, 2 human cases (total 4) were reported in 1968 and 1982 (Beaver *et al*., 1984), and since then, 28 additional cases were described until 2013 (Hernández-Chea *et al*., 2017). In Ecuador, as early as in 1922, a paragonimiasis patient was found from a coastal region of Chone-Manabi (Calvopiña *et al*., 2014); this seems to be the first human case infected with *P. mexicanus*. Thereafter, until 2007, the total number of human infections in Ecuador was 3822 patients (Calvopiña *et al*., 2014). Using a combination of ITS2, 28S and *cox*1 sequences, the phylogenetic position of *P. mexicanus* was analysed, and it was closest to *P. heteretremus* and then *P. macrorchis*; however, far from *P. westermani* and *P. siamensis* (Devi *et al*., 2013). However, in Mexico, Ecuador (López-Caballero *et al*., 2013) and Guatemala (Landaverde-González *et al*., 2022), several new genetic groups distinct from *P. mexicanus* (origin; Colima, Mexico) were found based on *cox*1 sequences. One from Ecuador could be assigned as *P. ecuadoriensis* Voelker and Arzube, 1979 (once synonymized with *P. mexicanus* by Vieira *et al*. in 1992), and two possible new species (or subspecies) groups included one from Chiapas, Mexico and San José, Guatemala and the other from Veracruz, Mexico (Iwagami *et al*., 2003; López-Caballero *et al*., 2013). Freshwater crabs, *Hypolobocera aequatorialis, H. chilensis* and *H. guayaquilensis*, *Pseudothelphusa dilatata*, *P. nayaritae, P. propinqua* and *P. terrestris* carry the metacercariae (Blair *et al*., 1999a; Calvopiña *et al*., 2018). Raw crabs with vegetables and lemon juice is the main infection source for Peruvians (Nakamura-Uchiyama *et al*., 2002), and ceviche that contains uncooked crustacean meat is an important source for Mexicans (Procop, 2009). Clinically *P. mexicanus* infection is mostly of the pulmonary type (99.7% of 3822 cases in Ecuador) (Calvopiña *et al*., 2014). However, cerebral infection with intracerebral haemorrhage can occur (Brenes Madrigal *et al*., 1982).

#### Paragonimus skrjabini

*Paragonimus skrjabini* Chen, 1959 (syn. *P. szechuanensis, P. hueitungensis* and *P. veocularis*) was first described from the lungs of viverrid cats in Guangzhou, China (Blair *et al*., 1999a). Phylogenetic studies revealed that there exists a *P. skrjabini* complex, which includes *P. skrjabini, P. skrjabini miyazakii* and *P. proliferus* (syn. *P. hocuoensis*) (Doanh *et al*., 2013b; Yang *et al*., 2021; Shu *et al*., 2021*a*). *P. skrjabini* is now known to occur in China, Thailand, Vietnam and northeast India (Singh *et al*., 2006; Doanh *et al*., 2013b; Blair, 2019). Human infections were reported for the first time in Sichuan and then in Hunan (Blair *et al*., 1999a). Numerous provinces in China have been found to be endemic for *P. skrjabini* infection (Zhou *et al*., 2021). The first intermediate host is freshwater snails, including *Assiminea lutea, Tricula* spp. and *Neotricula* spp., and the second host is freshwater crabs, *Aprapotamon grahami, Isolapotamon* spp., *Sinopotamon* spp., *Tenuilapotamon* spp., *Stigmatomma denticulatum* and *Haberma nanum* (Blair *et al*., 1999a; Shu *et al*., 2021b). Morphologically this species is characterized by profusely branched ovary and testes and an elongated body (Blair *et al*., 2005; Narain *et al*., 2010). *P. skrjabini* more frequently cause cutaneous or cerebral infections than pulmonary lesions, and the skin lesions usually contain juvenile flukes; thus, humans are considered an abnormal definitive host (Nakamura-Uchiyama *et al*., 2002). Diagnosis is most commonly done by serological tests, including intradermal test and ELISA (Yu *et al*., 2017).

#### Paragonimus skrjabini miyazakii

*Paragonimus skrjabini miyazakii* (Kamo, Nishida, Hatsushika and Tomimura, 1961) Blair *et al*., 2005 (syn. *P. miyazakii*) was first described from dogs experimentally fed the metacercariae from a freshwater crab in Japan (Kamo *et al*., 1961). Human infections were first identified in the Kanton District of Honshu, Japan (Yokogawa *et al*., 1974). This lung fluke has been found in Shikoku, Kyushu, and the southern half of Honshu, but never from outside of Japan (Miyazaki, 1991). In the University of Miyazaki, Japan, approximately 800 cases (17–49 cases annually) of human paragonimiasis (predominantly by *P. westermani* and less frequently by *P. skrjabini miyazakii*) were diagnosed between 1986 and 2018 (Yoshida *et al*., 2019). Yatera *et al*. (2015) reviewed 46 *P. skrjabini miyazakii* patients reported from 1974 to 2009 having pulmonary involvement. Phylogenetic studies revealed that *P. skrjabini miyazakii* is clearly included among the *P. skrjabini* complex (Blair *et al*., 2005; Doanh *et al*., 2013b; Yang *et al*., 2021; Shu *et al*., 2021*a*; 2021*b*). Freshwater crabs, *Geothelphusa dehaani* carry the metacercariae (Blair *et al*., 1999a). The adult flukes are somewhat elongated with severely branched ovary and testes (Miyazaki, 1991; Blair *et al*., 2005). Whereas the main clinical features of *P. skrjabini miyazakii* infection are pleural manifestations with pneumothorax and pleural effusion (in most cases worms cannot mature to become adults in humans), those of *P. westermani* infection are pulmonary involvement (Yatera *et al*., 2015).

#### Paragonimus uterobilateralis

*Paragonimus uterobilateralis* Voelker and Vogel, 1965 was originally described from the mongoose in Cameroon (Narain *et al*., 2010), and is now known to be distributed in mid-western sub-Saharan Africa (Blair *et al*., 1999a). Human infection was first found in Nigeria (Blair *et al*., 1999a). Up to 2008, there had been 2295 confirmed cases of human paragonimiasis in Africa, which was mostly due to *P. africanus* and *P. uterobilateralis*; the vast majority of these cases occurred in Nigeria and Cameroon (Aka *et al*., 2008; Cumberlidge *et al*., 2018). Freshwater crabs, *Liberonautes* spp. and *Sudanonautes* spp., are the second intermediate hosts (Blair *et al*., 1999a). This lung fluke is morphologically similar to *P. africanus* but differs in having similar-sized oral and ventral suckers (Miyazaki, 1991). It has a delicately branched ovary; however, it has moderately branched testes larger than the ovary (Blair *et al*., 1999a). Uncooked crab meat is an important source of human infection (Aka *et al*., 2008). Clinical manifestations are similar to *P. westermani* infection. Cerebral infection has been suspected but never proved (Aka *et al*., 2008).

## Intestinal flukes

Intestinal flukes are taxonomically diverse, including three large groups, namely, heterophyids (family Heterophyidae), echinostomes (family Echinostomatidae), and other groups, including amphistomes, brachylaimids, cyathocotylids, diplostomes, fasciolids, gymnophallids, isoparorchiids, lecithodendriid-like flukes, microphallids, nanophyetids, plagiorchiids and strigeids (Yamaguti, 1971; Chai, 2019; Chai and Jung, 2020). At least 75 different species have been described which infect an estimated 40–50 million people (Chai *et al*., 2009; Chai and Jung, 2020). Among them, heterophyids include 28 species (14 species are with more than 10 human cases; Table 4), echinostomes include 24 species (16 species; Table 5), and other groups are comprised of 23 species (10 species; Table 6). In this section, 40 species having more than 10 human cases are briefly introduced in the text, and the other 35 miscellaneous species are briefly mentioned in Supplementary Table. Molecular techniques using ITS and *cox*1 genes have been applied to differentiate eggs, larvae, as well as adults of various heterophyid species (Duflot *et al*., 2021). Intestinal flukes are easily treated with 10 mg kg^−1^ single dose of praziquantel or 2 g single dose of niclosamide (Table 2).

**Table 4.** Heterophyid intestinal flukes infecting humans with their life cycle and geographical distribution^a^

Species of heterophyids	First intermediate host (snail)	Second intermediate host	Reservoir host	Geographical distribution
*Metagonimus yokogawai*	*Semisulcospira* sp.	Freshwater fish (sweetfish, dace)	Dog, cat, rat, fox, kite	China, India, Japan, South Korea, Taiwan, Russia
*Metagonimus miyatai*	*Semisulcospira* sp.	Freshwater fish (chub, minnow)	Dog, red fox, raccoon, kite	Japan, South Korea
*Metagonimus takahashii*	*Semisulcospira* sp. *Koreanomelania* sp.	Freshwater fish (carp)	Dog, cat, kite, pelican	Japan, South Korea
*Heterophyes heterophyes*	*Pirenella conica*	Brackish water fish (mullet, goby, tilapia)	Dog, fox, jackal	Egypt, Greece, India, Iran, Israel, Italy, Kuwait, Sri Lanka, Saudi Arabia, Spain, Sudan, Thailand, Tunisia, Turkey, United Arab Emirate, Yemen
*Heterophyes nocens*	*Cerithidea* sp.	Brackish water fish (mullet, goby)	Dog, cat	China, Japan, Korea, Thailand (?)
*Haplorchis taichui*	*Melania* sp. *Melanoides* sp.	Freshwater fish (carp, minnow)	Dog, cat, bird	Bangladesh, China, Egypt, India, Iraq, Israel, Kuwait, Lao PDR, Malaysia, Myanmar, The Philippines, Sri Lanka, Taiwan, Thailand, USA (Hawaii), Vietnam
*Haplorchis pumilio*	*Melania* sp.	Cyprinoid fish silurid fish cobitid fish	Dog, cat	Australia, Cambodia, China, Egypt, India, Iraq, Israel, Kenya, Lao PDR, Malaysia, Mexico, Myanmar, Peru, The Philippines, Sri Lanka, Taiwan, Thailand, USA, Venezuela, Vietnam
*Haplorchis yokogawai*	*Melanoides* sp.	Freshwater fish (carp, loach) brackish water fish (mullet)	Dog, cat, cattle	Australia, Cambodia, China, Egypt, India, Indonesia, Kuwait, Lao PDR, Malaysia, Myanmar, The Philippines, Sri Lanka, Taiwan, Thailand, USA (Hawaii), Vietnam
*Centrocestus formosanus*	*Stenomelania* sp.	Cyprinoid fish	Dog, fox, chicken, duck	Brazil, China, Colombia, Costa Rica, Croatia, Egypt, India, Japan, Lao PDR, Mexico, The Philippines, Taiwan, Thailand, Tunisia, Turkey, USA, Vietnam
*Heterophyopsis continua*	Unknown	Brackish water fish (perch, goby, shad)	Cat, duck, sea-gull	China, Japan, Korea, Saudi Arabia, Thailand, United Arab Emirates, Vietnam
*Pygidiopsis summa*	*Cerithidea* sp.	Brackish water fish (mullet, goby)	Cat	South Korea, Japan, Vietnam
*Pygidiopsis genata*	*Melanoides* sp.	Brackish water fish (mullet, tilapia)	Dog, cat, rat, fox, wolf, shrew, kite, pelican, duck	Egypt, Iran, Israel, Kuwait, Romania, The Philippines, Tunisia, Ukraine
*Stellantchasmus falcatus*	*Stenomelania* sp.	Brackish water fish (mullet)	Dog, cat	Australia, China, India, Iran, Israel, Japan, Korea, Lao PDR, The Philippines, Taiwan, Thailand,USA (Hawaii), Vietnam
*Stictodora fuscata*	Unknown	Freshwater and brackish water fish (carp, goby)	Dog, cat	South Korea, Japan, Kuwait

a The geographical distributions of heterophyids are referred from Chai (2019) and Chai and Jung (2019).

**Table 5.** Echinostomes infecting humans with their life cycle and geographical distribution

Species of echinostomes	First intermediate host (snail)	Second intermediate host	Reservoir host	Geographical distribution^a^
*Echinostoma revolutum*	*Lymnaea* sp. *Physa* sp. *Segmentina* sp.	Freshwater snail, clam, tadpole	Dog, cat, rat, muskrat, duck, goose	*Asia* (Bangladesh, Cambodia, China, India, Indonesia, Japan, Korea, Lao PDR, Malaysia, Taiwan, Thailand, Vietnam), *Europe* (Austria, Belarus, Bulgaria, Czech Rep., England, Finland, France, Germany, Greece, Hungary, Iceland, The Netherlands, Poland, Russia, Slovak Rep., Yugoslavia), *The Middle East* (Iran), *Oceania* (New Zealand), *North America* (USA), *South America* (Brazil)
*Echinostoma cinetorchis*	*Hippeutis cantori* *Segmentina hemisphaerula*	Freshwater snail, fish (loach), tadpole	Rat	South Korea, Japan, China, Taiwan, Vietnam
*Echinostoma ilocanum*	*Gyraulus* sp. *Hippeutis* sp.	Large snails (*Viviparus* sp., *Pila* sp.)	Dog, rat	The Philippines, Cambodia, China, India, Indonesia, Lao PDR, Malaysia, Thailand
*Echinostoma lindoense*	*Anisus* spp.	*Lymnaea, Planorbis, Gyraulus, Viviparus, Biomphalaria* spp.	Bird, mammal	Indonesia, Malaysia, Thailand, The Philippines
*Echinostoma mekongi*	Unknown	Freshwater snail (*Filopaludina* sp.)	Unknown	Cambodia (probably also in Lao PDR, Thailand, Vietnam)
*Isthmiophora hortensis*	*Lymnaea* sp. *Radix* sp.	Freshwater fish (loach, etc.)	Dog, cat, rat	Japan, South Korea, China
*Echinochasmus japonicus*	*Parafossarulus* sp.	Cyprinoid fish	Cat, duck, egret	Japan, South Korea, China, Kuwait, Lao PDR, Russia, Taiwan, Thailand, Vietnam
*Echinochasmus perfoliatus*	*Parafossarulus* sp. *Bithynia* sp. *Lymnaea* sp.	Freshwater fish (carp, chub)	Dog, cat, rat, fox, wild boar, fowl	Bulgaria, China, Croatia, Denmark, Egypt, England, Hungary, India, Italy, Japan, Korea, Poland, Romania, Russia, Serbia, Taiwan, Thailand, Ukraine, Vietnam
*Echinochasmus liliputanus*	*Parafossarulus* sp.	Freshwater fish (carp)	Dog, cat, fox, badger, raccoon	Egypt, China, Israel, Sri Lanka, Syria
*Echinochamus fujianensis*	*Bellamya* sp.	Freshwater fish (carp)	Dog, cat, rat, pig	China
*Echinochasmus caninus*	Unknown	Freshwater fish (*Macropodus* sp.)	Dog	India, China, Thailand, Vietnam, Lao PDR
*Artyfechinostomum malayanum*	*Indoplanorbis* sp. *Gyraulus* sp.	Large snails (*Pila, Gyraulus, Indoplanorbis* spp.)	Dog, cat, rat, pig, mouse, hamster, house-shrew	China, India, Indonesia, Lao PDR, Malaysia, The Philippines, Singapore, Thailand
*Artyfechinostomum sufrartyfex*	*Indoplanorbis* sp. *Lymnaea* sp.	Freshwater snail, fish, frog	Dog, rat, pig	India, Vietnam (?)
*Artyfechinostomum oraoni*	*Lymnaea* sp.	Unknown	Pig	India
*Hypoderaeum conoideum*	*Indoplanorbis* sp. *Planorbis* sp. *Lymnaea* sp.	Large snail, tadpole	Duck, goose, fowl	Bangladesh, China, Indonesia, Japan, Mexico, North America, Russia, Spain, Taiwan, Thailand
*Acanthoparyphium tyosenense*	Marine megagastropod marine gastropod	Brackish water bivalve, gastropod	Duck	South Korea, Japan

a The geographical distributions of these echinostomes are referred from Chai (2019) and Chai and Jung (2019).

**Table 6.** Other intestinal fluke species infecting humans with their life cycle and geographical distribution

Species of intestinal fluke	First intermediate host (snail)	Second intermediate host	Reservoir host	Geographical distribution^a^
*Brachylaima cribbi*	Land snail (*Theba pisana*)	Land snail (*Cernuella virgata*)	Bird, reptile, mammal	Australia
*Caprimorgorchis molenkampi*	*Bithynia* sp.	Naiad of dragonfly	Rat, bat	Cambodia, Indonesia, Lao PDR, Thailand
*Fasciolopsis buski*	*Segmentina* sp. *Hippeutis* sp. *Gyraulus* sp.	Aquatic plant (water chestnut, water bamboo, water caltrop, water hyacinth, root of lotus)	Dog, pig, rabbit	Bangladesh, Cambodia, China, India, Indonesia, Japan, Korea, Lao PDR, Malaysia, Myanmar, Nepal, Pakistan, The Philippines, Singapore, Taiwan, Thailand, Vietnam
*Gastrodiscoides hominis*	*Helicorbis coenosus*	Aquatic plant, tadpole, frog, crayfish	Pig, mouse deer, rat, monkey	Cambodia, China, England, India, Indonesia, Japan, Kazakhstan, Malaysia, Myanmar, Nigeria, Pakistan, The Philippines, Russia, Thailand, USA, Vietnam, Zambia
*Gymnophalloides seoi*	Unknown	Oyster (*Crassostrea gigas*)	Palearctic oystercatcher, cat	South Korea
*Microphallus brevicaeca*	Unknown	Brackish water crab (*Carcinus maenas*), shrimp (*Macrobrachium* sp.)	Bird, monkey, mammal	Papua New Guinea, The Philippines
*Nanophyetus salmincola*	*Oxytrema silicula*	Salmonid fish non-salmonid fish	Dog, cat, raccoon, fox, bird	Canada, USA
*Nanophyetus schikhobalowi*	*Semisulcospira* sp.	Salmonid fish non-salmonid fish	Rat, fox	Russia
*Neodiplostomum seoulense*	*Hippeutis cantori* *Segmentina hemisphaerula*	Tadpole, frog, snake (paratenic host)	Rat, mouse	South Korea, China
*Phaneropsolus bonnei*	*Bithynia* sp. (?)	Naiad of dragonfly	Monkey	Cambodia, India, Indonesia, Lao PDR, Malaysia, Thailand

a The geographical distributions of these intestinal flukes are referred from Chai (2019) and Chai and Jung (2019).

### Heterophyid species

#### Metagonimus *spp.*

*Metagonimus yokogawai* (Katsurada, 1912) Katsurada, 1912 was originally described from an experimentally infected dog in Taiwan and then reported mostly from Asian countries (Yu and Chai, 2013). Sequences of nuclear 28S rDNA and mitochondrial *cox*1 genes were used to discriminate this species from the related ones, *M. miyatai* and *M. takahashii* (Chai, 2019). The main clinical symptoms of *M. yokogawai* infection are mild-to-severe gastrointestinal trouble, including abdominal pain, diarrhoea and indigestion (Chai and Jung, 2020). The histopathology of the small intestine is characterized by villous atrophy and crypt hyperplasia accompanied by inflammatory reactions (Chai, 2019). The first case of human infection was discovered in Japan by Yokogawa S. in 1913, and thereafter numerous succeeding reports were published (Ito, 1964). The sweetfish *Plecoglossus altivelis*, dace *Tribolodon hokonensis* or *T. taczanowskii,* and perch *Lateolabrax japonicus* are the major fish intermediate host (Yu and Chai, 2013). The principal mode of human infection is ingestion of raw or improperly cooked fish (Chai *et al*., 2009; Chai and Jung, 2017). *M. yokogawai* is distributed mainly in the Far East and East Asia (Chai, 2019). In South Korea, endemic areas are scattered along almost all large and small rivers and streams in eastern and southern coastal areas (Chai and Lee, 2002; Chai *et al*., 2009). In Japan, the prevalence reported in humans has been generally lower than that in South Korea (Chai and Jung, 2020). In China, human infections were reported in Guangdong, Anhui, Hubei and Zhejiang Province (Yu and Mott, 1994). The diagnosis is based on the recovery of eggs in fecal examinations. In Russia, *Metagonimus* infection has been reported under the name *M. yokogawai* in the Amur and Ussuri valleys of the Khabarovsk Territory, and the prevalence was 20–70% among the ethnic minority group (Yu and Mott, 1994). Recently, however, the parasite in this area (obtained from experimental rats) was reported as a new species (*Metagonimus suifunensis*) based on molecular analyses (Shumenko *et al*., 2017).

*Metagonimus miyatai* Saito, Chai, Kim, Lee et Rim, 1997 was first found in Japan in 1941 but reported a long time later as a distinct species (Saito *et al*., 1997). Human infections seem to have existed in Japan but there had been no confirmed reports. In South Korea, human infections were first recognized along the Geum River in 1980 by detecting eggs in feces (Chai, 2019). Adult flukes were recovered from 32 people living along the Namhan River in Umsong and Yongwol County (Chai *et al*., 1993). Freshwater fish, including *Zacco platypus, Z. temminckii*, *P. altivelis, Tribolodon hakonensis* and *T. taczanowskii,* are the second intermediate hosts (Yu and Chai, 2013). In Japan, fish from small rivers in the Shizuoka Prefecture were found to have *M. miyatai* infection (Kino *et al*., 2006).

*Metagonimus takahashii* (Takahashi, 1929) Suzuki, 1930 was originally described in Japan and now known to exist also in South Korea (Yu and Chai, 2013). The crussian carp *C. carassius*, carp *C. carpio*, dace *T. taczanowskii* and perch *L. japonicus* carry the metacercariae (Yu and Chai, 2013). In Japan, this fluke was reported in Okayama, Hiroshima and around the Lake Biwa (Ito, 1964; Urabe, 2003). In South Korea, human infections were first identified from riparian people along the Hongcheon River, Gangwon-do (Province) (Ahn and Ryang, 1988). Subsequently, an endemic focus was discovered in 1993 from Umsong County along the upper reaches of the Namhan River (Chai *et al*., 1993).

#### Heterophyes heterophyes *and* H. nocens

*Heterophyes heterophyes* (*v.* Siebold, 1852) Stiles and Hassall, 1900 was first described based on specimens obtained at autopsy of an Egyptian, and the Nile Delta of Egypt and Sudan is now known to be important endemic areas (Yu and Mott, 1994; Chai, 2007). The distribution of *H. heterophyes* is also known in Europe, the Middle East and North Africa (Yu and Mott, 1994; Chai, 2007). Sequences of nuclear ITS2 and 28S rDNA and mitochondrial *cox*1 have been used to differentiate *Heterophyes* species (Chai, 2019). Brackish water fish, including the mullet *Mugil cephalus* and tilapia *Tilapia nilotica,* are the second intermediate hosts (Chai, 2007). Humans are infected by eating raw or inadequately cooked brackish water fish.

*Heterophyes nocens* Onji and Nishio, 1916 was first described from experimental dogs and cats fed mullets in Japan (Chai, 2007). This fluke has been reported also in South Korea and Japan (Chai *et al*., 2009; Chai and Jung, 2017). Brackish water fish, including the mullet and goby *Acanthogobius flavimanus*, are the second intermediate hosts (Guk *et al*., 2007). Humans are infected by consuming raw or inadequately cooked brackish water fish. In South Korea, residents of southwestern coastal areas and islands showed 10–70% egg-positive rates (Chai *et al*., 2004, 2009). In Japan, Kochi, Chiba, Yamaguchi, Chugoku, Hiroshima and Shizuoka Prefectures were the places where human infections were recorded (Kino *et al*., 2002). Diagnosis is based on the recovery of eggs in fecal examinations, but eggs should be differentiated from those of other heterophyids and small liver flukes.

#### Haplorchis *spp.*

*Haplorchis taichui* (Nishigori, 1924) Chen, 1936 was originally described from birds and mammals in Taiwan (Chai, 2007; Chai and Jung, 2017). The first report of human infections was published from the Philippines (Africa *et al*., 1940). Now this fluke is known to be distributed widely in Asia and the Middle East (Chai *et al*., 2007, 2009, 2012*a*; Sohn *et al*., 2014). PCR targeting ITS1, ITS2 and *cox*1 has been proved to be useful for genetic studies of *Haplorchis* species and opisthorchiids (Chai, 2019). In Laos, hyperendemic areas with average individual worm loads of 12 079 (Champasak Province) and 21 565 specimens (Saravane Province) were reported (Chai *et al*., 2013a). Freshwater fish, *Cyprinus* spp., *Gambusia affinis, Hampala dispar* and *Puntius* spp., harbour the metacercariae (Chai *et al*., 2009).

*Haplorchis pumilio* (Looss, 1896) Looss, 1899 was first recorded from birds and mammals in Egypt (Chai *et al*., 2009). Later, in Taiwan, a successful experimental infection of a human volunteer was reported in 1924 (Chai *et al*., 2009). Numerous human infections were found thereafter, and now this fluke is known to be distributed widely from Africa to Asia, Oceania and the Americas (Chai *et al*., 2009, 2012*a*, 2014, 2015; Chung *et al*., 2011). The second intermediate host is freshwater fish of the Cyprinidae, Siluridae and Cobitidae (Chai *et al*., 2009).

*Haplorchis yokogawai* (Katsuta, 1932) Chen, 1936 was first reported from dogs and cats experimentally fed mullets in Taiwan (Chai *et al*., 2009). An experimental human infection was successful in Taiwan but natural human infections were reported for the first time in the Philippines (Africa *et al*., 1940). This fluke is now known to be distributed in Asia, Australia and Egypt (Chai *et al*., 2007, 2009, 2014). Freshwater fish *Cyclocheilichthys armatus, Hampala dispar, Misgurnus* sp., *Mugil* spp. and *Puntius* spp. harbour the metacercariae (Chai *et al*., 2009).

#### Centrocestus formosanus

*Centrocestus formosanus* (Nishigori, 1924) Price, 1932 (syn. *Centrocestus caninus*) was first found in a dog experimentally infected with metacercariae from freshwater fish in Taiwan (Chai *et al*., 2009; Chai and Jung, 2017). A successful experimental infection of a human volunteer was reported at the same time (Chai *et al*., 2009; Chai and Jung, 2017). Two human infections reported in Thailand under the name *C. caninus* (Waikagul *et al*., 1997) are now considered as *C. formosanus* infection. In Laos, 7 human infections were detected (Chai *et al*., 2013*b*). Now this fluke is distributed almost all over the world, including Asia, Europe and North and South America (Chai *et al*., 2009, 2012*a*, 2013*b*, 2014; Wanlop *et al*., 2017). Human infections were reported from China, Taiwan, the Philippines, Thailand, Laos and Vietnam (Yu and Mott, 1994; Chai *et al*., 2009; Chai, 2019). The freshwater fish, including *Cyclocheilichthys repasson* and *Puntius brevis,* are the second host (Chai *et al*., 2009).

#### Heterophyopsis continua

*Heterophyopsis continua* (Onji and Nishio, 1916) Price, 1940 was originally described from experimental cats infected with metacercariae in the mullet in Japan. Without proper literature background, the presence of human infections was mentioned in Japan (Yamaguti, 1958). In South Korea, adult flukes were recovered from more than 10 human cases (Chai, 2019). The second intermediate host includes the perch *L. japonicus,* goby *A. flavimanus,* shad *Clupanodon punctatus*, conger eel *Conger myriaster*, and sweetfish *P. altivelis* (Chai *et al*., 2009). This fluke is distributed in Asia and the Middle East (Chai *et al*., 2012a; Chai, 2019).

#### Pygidiopsis summa *and* P. genata

*Pygidiopsis summa* Onji and Nishio, 1916 was originally described from dogs experimentally infected with metacercariae from brackish water fish in Japan and thereafter this fluke was reported also from South Korea (Chai *et al*., 2009; Chai and Jung, 2017). In Japan, human infections were suggested by the recovery of eggs in feces in 1929, and adult flukes were subsequently isolated in 1965 (Chai *et al*., 2009). In South Korea, human infections (8 cases) were first discovered from a salt-farm village in a western costal area of Jeollabuk-do (Province) (Seo *et al*., 1981). Now this fluke is known to be distributed on many western and southern coastal islands of South Korea (Chai *et al*., 2004). Its presence was also reported in Vietnam (Vo *et al*., 2008). Sequences of 28S rDNA and *cox*1 were used to assess the phylogenetic relationships of *P. summa* with *C. sinensis, M. yokogawai* and *M. takahashii* (Chai, 2019).

*Pygidiopsis genata* Looss, 1907 was originally described from a pelican in Egypt, and then also found in pelicans in Romania, experimental dogs and cats in the Philippines, a Persian wolf (in Berlin Museum), and dogs and cats in Israel (Palestine) (Witenberg, 1929; Africa *et al*., 1940). This parasite is distributed in Egypt, the Middle East and the Philippines (Chai and Jung, 2020). Human infections were first found in Egypt (Youssef *et al*., 1987a). Brackish water fish, including tilapia, mullet and *Barbus canis*, are the second intermediate hosts (Witenberg, 1929; Youssef *et al*., 1987b).

#### Stellantchasmus falcatus

*Stellantchasmus falcatus* Onji and Nishio, 1916 was first described from cats experimentally fed the mullet harbouring the metacercariae in Japan (Chai *et al*., 2009). Human infections were first reported in Japan and thereafter in many Asian-Pacific countries, including South Korea, the Philippines, Hawaii, Thailand and Vietnam (Chai *et al*., 2009, 2012*a*, 2016).

#### Stictodora fuscata

*Stictodora fuscata* (Onji and Nishio, 1916) Yamaguti, 1958 was first discovered from experimental cats fed infected mullets in Japan (Chai and Jung, 2017). Human infection was first found in South Korea in a young man who regularly consumed mullets and gobies raw (Chai *et al*., 1988). Subsequently, 13 additional cases were detected in a southwestern coastal area (Chai and Lee, 2002). Gobies *A. flavimanus* and topmouth gudgeons *Pseudorasbora parva* are the fish hosts (Yamaguti, 1958; Chai *et al*., 2009).

#### Miscellaneous heterophyid species

Fourteen species of miscellaneous heterophyid flukes having less than 10 human infection cases are briefly introduced in Supplementary Table. See Chai and Jung (2020) for details.

## Echinostome species

### Echinostoma *spp.*

*Echinostoma revolutum* (Froelich, 1802) Dietz, 1909 is the oldest echinostome species ever recorded in the literature (Chai, 2009; Chai *et al*., 2020). It was originally described in 1798 from a wild duck *Anas boschas fereae* naturally infected in Germany (Kanev, 1994). This species is now known to be distributed widely in Asia, Europe, Africa, Oceania and North and South America (Chai *et al*., 2009). The first report of human infection was published from Taiwan in 1929, where its prevalence among people was 2.8–6.5% (Yu and Mott, 1994). Subsequently, human infections were reported in China, Indonesia, Thailand and Russia (Chai *et al*., 2009). The flukes can cause gastrointestinal trouble, mucosal ulceration and bleeding (Chai, 2009). Tadpoles, gastropod snails (*Physa occidentalis, Lymnaea* sp. and *Filopaludina* sp.) and clams (*Corbicula producta*) can be the second intermediate hosts (Beaver *et al*., 1984; Yu and Mott, 1994; Chai *et al*., 2011; Chantima *et al*., 2013).

*Echinostoma cinetorchis* Ando and Ozaki, 1923 was first discovered from naturally infected rats in Japan (Chai *et al*., 2009). Adult flukes of this echinostome species are morphologically characterized by having a reduced number (none or 1, rarely 2) and abnormal location of testes (Chai, 2009). Human infections were first documented in Japan, and subsequently also detected in South Korea and China (Chai *et al*., 2009).

*Echinostoma ilocanum* (Garrison, 1908) Odhner, 1911 was first described from 5 prisoners in Manila, the Philippines (Chai *et al*., 2009). Subsequently, numerous human infection cases were discovered in Asian countries (Sohn *et al*., 2011; Chai *et al*., 2018). For example, in northern Luzon, the Philippines, the prevalence among the people was high, in the range from 7% to 17% (Chai *et al*., 2009). In Oddar Meanchey Province, Cambodia, the general population and the student group revealed 1.8% and 0.7% egg-positive rates, respectively (Sohn *et al*., 2011). Large snails, *Pila conica* (the Philippines) and *Viviparus javanicus* (Java) are the second intermediate hosts (Beaver *et al*., 1984; Yu and Mott, 1994).

*Echinostoma lindoense* Sandground and Bonne, 1940 was described from human population in the Lake Lindoe region of Central Celebes, Indonesia (Chai, 2019). A high prevalence (24–96%) of human infection was reported in Indonesia (Chai *et al*., 2009). It is now known to be distributed in South Asian countries (Chai, 2009).

*Echinostoma mekongi* Cho, Jung, Chang, Sohn, Sinuon and Chai, 2020 was described as a new species based on adult flukes recovered from 6 humans residing along the Mekong River in Kratie and Takeo Province, Cambodia (Cho *et al*., 2020). Further human cases (more than 10) were found in Kandal Province, Cambodia (unpublished data). The freshwater snails, *Filopaludina martensi cambodjiensis*, from Pursat Province, Cambodia were found to carry the metacercariae (Chai *et al*., 2021b). This echinostome seems to exist in neighbouring countries, such as Laos, Thailand and Vietnam.

### Isthmiophora hortensis

*Isthmiophora hortensis* (Asada, 1926) Kostadinova and Gibson, 2002 (syn. *Echinostoma hortense*) was first found from rats in Japan, and then also from rats in South Korea and China (Chai and Jung, 2019). Human infections were first documented in Japan in 1976 and then also found in South Korea and China (Chai and Lee, 2002; Chai *et al*., 2009). In South Korea, clinical cases with significant abdominal symptoms were diagnosed by extracting living worms by gastroduodenal endoscopy (Chai *et al*., 2009). Loaches, *Misgurnus anguillicaudatus* and *M. mizolepis*, and other freshwater fish, including *Odontobutis obscura interrupta* and *Moroco oxycephalus*, are the second intermediate hosts (Chai *et al*., 2009).

### Echinochasmus *spp.*

*Echinochasmus japonicus* Tanabe, 1926 was originally reported in Japan from dogs, cats, rats, mice and birds experimentally fed the metacercariae encysted in freshwater fish (Chai and Jung, 2019). This fluke is now known to occur mainly in Far Eastern countries (Chai and Lee, 2002; Chai and Jung, 2019). In Japan, an experimental human infection was reported to be successful, and in China and South Korea natural human infections were discovered (Chai *et al*., 2009). Freshwater fish, including *P. parva*, *Hypomesus olidus* and *Gnathopogon strigatus*, are the second intermediate hosts (Chai *et al*., 1985; Choi *et al*., 2006).

*Echinochasmus perfoliatus* (Ratz, 1908) Gedoelst, 1911 was originally reported from dogs in Romania, and then found again in dogs and cats in Hungary (Chai *et al*., 2009). Now its distribution is known to be wide from Asia to Europe (Chai *et al*., 2009). Human infections were reported in Japan (Chai *et al*., 2009). In Guangdong, Fujian, Anhui and Hubei Provinces of China, 1.8% prevalence was reported among people (Yu and Mott, 1994). Freshwater fish, including *Carassius* sp., *Zacco platypus, Z. temminckii,* and *P. parva*, are the second intermediate hosts (Rim, 1982*c*; Yu and Mott, 1994).

*Echinochasmus liliputanus* (Looss, 1896) Odhner, 1910 was found from dogs, cats, and birds in Egypt, Syria and Israel (Palestine) (Yamaguti, 1958). The first human infections, with a high prevalence of 13.4% among 2426 people, were reported in Anhui Province, China in 1991 (Yu and Mott, 1994). Since that time, more than 2500 human cases had been reported in Anhui Province, China (Xiao *et al*., 2005). *P. parva* and goldfish are the second intermediate hosts (Xiao *et al*., 2005). The mode of human infections is consumption of raw or improperly cooked fish or drinking untreated water containing the cercariae (Chai *et al*., 2009).

*Echinochasmus fujianensis* Cheng, Lin, Chen, *et al*., 1992 was originally reported from humans, dogs, cats, pigs and rats in Fujian Province, China (Yu and Mott, 1994). The prevalence among residents (mostly children) in Fujian Province ranged 1.6–7.8% (Yu and Mott, 1994). *P. parva* and *Cyprinus carpio* are the second hosts (Yu and Mott, 1994).

*Echinochasmus caninus* (Verma, 1935) Chai *et al*., 2019 (syn. *Episthochasmus caninum, Episthmium caninum*) was first described from dogs in India. Its metacercariae were found in fish *Macropodus opercularis,* and adult flukes were detected in dogs in Hainan Island, China (Chai *et al*., 2019b). Human infections were reported in northeastern Thailand and Lao PDR (Chai *et al*., 2019b).

### Artyfechinostomum *spp.*

*Artyfechinostomum malayanum* (Leiper, 1911) Mendheim, 1943 (syn. *Echinostoma malayanum*) was originally reported from a human in Malaysia (Beaver *et al*., 1984), and subsequently found in other Asian countries (Chai *et al*., 2009, 2012*b*; Sohn *et al*., 2017). Large snails, i.e. *I. exustus, A. convexiusculus* and *Pila scutate*, play the role of the second intermediate hosts (Yu and Mott, 1994; Belizario *et al*., 2007).

*Artyfechinostomum sufrartyfex* Lane, 1915 (syn. *Artyfechinostomum mehrai*) was originally discovered from an Assamese girl in India (Yu and Mott, 1994) and later in Vietnam (Tran *et al*., 2016). Freshwater snails, fish or frogs harbour the metacercariae (Raghunathan and Srinivasan, 1962). Further human cases were reported in India (Chai and Jung, 2020).

*Artyfechinostomum oraoni* Bandyopadhyay, Manna et Nandy, 1989 was originally reported from 20 human infection cases in a tribal community near Calcutta, India (Bandyopadhyay *et al*., 1989, 1995). The life cycle is to be determined. This fluke may provoke fatal diarrhoea in pigs (Bandyopadhyay *et al*., 1989).

### Hypoderaeum conoideum

*Hypoderaeum conoideum* (Bloch, 1872) Dietz, 1909 was originally reported from various species of birds in Europe (Yamaguti, 1958). This echinostome is now known to be distributed in Europe, Asia and Siberia (Yamaguti, 1958; Rim, 1982c; Beaver *et al*., 1984). The first human infections were reported in northeastern Thailand; the prevalence among 254 residents was 55% (Yokogawa *et al*., 1965). Large snails and tadpoles play the role of the second intermediate hosts (Chai *et al*., 2009).

### Acanthoparyphium tyosenense

*Acanthoparyphium tyosenense* Yamaguti, 1939 was originally reported from the duck *Melanitta fusca stejnegeri* and *M. nigra americana* caught in South Korea (Chai, 2009; Chai and Jung, 2019). Its geographical distribution is confined to South Korea and Japan (Kim *et al*., 2004). Humans infected with this echinostome were first discovered in coastal villages of Jeollabuk-do (Province), South Korea (Chai *et al*., 2001). Brackish water bivalves, including *Mactra veneriformis* and *Solen* spp*.,* and brackish water gastropod *Neverita bicolor,* were found to harbour the metacercariae (Chai *et al*., 2001; Kim *et al*., 2004).

### Miscellaneous echinostome species

Eight species of miscellaneous echinostome flukes having less than 10 human infection cases are briefly introduced in Supplementary Table. See Chai and Jung (2020) for details.

## Other intestinal fluke species

### Brachylaima cribbi

*Brachylaima cribbi* Butcher and Grove, 2001 was first described by Butcher and Grove (2001) in Australia. Human infections (about 15 cases) were reported in Australia (Butcher *et al*., 1998, 2003). Helicid land snails, such as, *Cernuella virgata*, serve as the source of human infection (Butcher and Grove, 2005). Clinical symptoms include diarrhoea, abdominal pain, low-grade fever and fatigue (Butcher *et al*., 2003).

### Caprimolgorchis molenkampi

*Caprimolgorchis molenkampi* (Lie, 1951) Baugh, 1957 (syn. *Prosthodendrium molenkampi*) was originally reported from 2 human autopsies in Indonesia (Manning and Lertprasert, 1973). Later, this fluke was found again in 14 human autopsies in northeastern Thailand (Manning *et al*., 1970; Manning and Lertprasert, 1973). Currently, this fluke shows a considerable prevalence in northeast Thailand and Laos (Chai *et al*., 2009). Naiads and adults of dragon- and damselflies serve as the second intermediate hosts (Manning and Lertprasert, 1973).

### Fasciolopsis buski

*Fasciolopsis buski* (Lankester, 1857) Odhner, 1902 was originally described in 1843 based on the worms in the duodenum of an Indian sailor (Beaver *et al*., 1984; Mas-Coma *et al*., 2005; Tandon *et al*., 2013). Now this fluke is known to be a common intestinal parasite of humans and pigs in Asia (Chai *et al*., 2009; Tandon *et al*., 2013). The estimated number of Asian populations infected with *F. buski* is about 10 million (Tandon *et al*., 2013). The prevalence ranged from 0.04% in Cambodia to 8.6–50% in Bangladesh, 25–61% in Taiwan and up to 85% in some areas of China (Tandon *et al*., 2013). Metacercariae can attach to the surface of aquatic plants, such as, water chestnut, water caltrop, water hyacinth, roots of the lotus, water bamboo and other aquatic vegetations; they may also float on the water (Beaver *et al*., 1984; Yu and Mott, 1994; Tandon *et al*., 2013). The main mode of human infection is the consumption of raw or improperly cooked aquatic plants, or peeling off the hull or skin of the plants by mouth before eating the raw nut (Yu and Mott, 1994).

### Gastrodiscoides hominis

*Gastrodiscoides hominis* (Lewis and McConnell, 1876) Leiper, 1913 was first found from an Indian patient (Beaver *et al*., 1984). This fluke is now known to be a common parasite of humans and pigs in various countries of Asia and Africa (Mas-Coma *et al*., 2005). Adult worms attach to the caecum and ascending colon of humans and may produce mucous diarrhoea (Beaver *et al*., 1984). The cercariae encyst on aquatic plants, or in tadpoles, frogs and crayfish (Yu and Mott, 1994).

### Gymnophallloides seoi

*Gymnophalloides seoi* Lee, Chai et Hong, 1993 was originally reported from a woman suffering from acute pancreatitis and gastrointestinal discomfort in South Korea (Lee *et al*., 1993; Chai *et al*., 2003). Subsequently, a southwestern coastal island was found to be a highly endemic area with 49% prevalence and heavy worm loads (Chai *et al*., 2003). This fluke is now known to be distributed in 25 seashore villages of western and southern coastal islands and 3 coastal inlands of South Korea (Chai *et al*., 2009). Oysters, *Crassostrea gigas* were verified to be the second intermediate hosts (Chai *et al*., 2003). Consumption of raw oysters is the main mode of human infection.

### Microphallus brevicaeca

*Microphallus brevicaeca* (Africa and Garcia, 1935) Baer, 1943 (syn. *Spelotrema brevicaeca*) was first described from birds and subsequently also found from 12 human autopsies in the Philippines (Africa *et al*., 1940). Eggs were shown to cause acute cardiac dilatation and egg granuloma in the heart, brain and spinal cord, especially in immunocompromised patients (Africa *et al*., 1940). Brackish water crabs *Carcinus maenas* and shrimps *Macrobrachium* sp. serve as the second intermediate hosts (Beaver *et al*., 1984).

### Nanophyetus salmincola *and* N. schikhobalowi

*Nanophyetus salmincola* (Chapin, 1926) Chapin in Hall, 1927 (syn. *Troglotrema salmincola*) was first found in dogs suffering from a fatal disease after the ingestion of uncooked salmon in the Pacific Coast of North America (Witenberg, 1932). Since 1974, at least 20 human infections were found in the USA (Witenberg, 1932; Eastburn *et al*., 1987). *N. salmincola* has been proven to be the vector of a rickettsia *Neorickettsia helmintheca* (Chai and Jung, 2020). This rickettsial infection can cause a serious and often fatal systemic disease known as salmon poisoning in animals such as dogs and foxes (Chai and Jung, 2020). However, salmon poisoning has never been reported in humans (Chai and Jung, 2020).

*Nanophyetus schikhobalowi* Skrjabin and Podjapolskaja, 1931 was discovered from native people in far eastern Siberia (Skrjabin and Podjapolskaja, 1931). It was described as a new species mainly because of their smaller egg size compared to *N. salmincola* (Skrjabin and Podjapolskaja, 1931). The far eastern part of Russia including Amur and Ussuri valleys of Khabarovsk Territory and north Sakhalin is an important endemic area, with an average prevalence of 5% (Yu and Mott, 1994).

### Neodiplostomum seoulense

*Neodiplostomum seoulense* (Seo, Rim et Lee, 1964) Hong and Shoop, 1995 was first found in house rats in South Korea (Seo, 1990). Its geographical distribution is confined to mountainous areas of South Korea (Seo, 1990) and a northeastern part of China (Chai *et al*., 2009). Adult flukes were recovered from a young man who suffered from acute abdominal pain and fever and had a history of consuming improperly cooked snakes (Seo, 1990). Experimental mice and rats showed severe mucosal damage in the intestinal tract with frequent host death and fecundity reduction (Lee *et al*., 1985; Chai *et al*., 2009; Shin *et al*., 2016). Military soldiers who had eaten raw snakes during their survival training were found to be infected with this fluke (Hong *et al*., 1984; Chai *et al*., 2009). Tadpoles and frogs of *Rana* sp. are the second intermediate hosts, and the snake *Rhabdophis tigrina* is a paratenic host (Seo, 1990).

### Phaneropsolus bonnei

*Phaneropsolus bonnei* Lie, 1951 was originally reported based on adult flukes recovered from a human autopsy in Indonesia, and subsequently this fluke was found in monkeys in Malaysia and India (Manning *et al*., 1970). Human infections with this fluke were first reported in 15 human autopsies in northeastern Thailand (Manning *et al*., 1970). Thailand and Laos are currently important countries where this fluke infection is considerably high among the people (Chai *et al*., 2009). Insects, particularly naiads and adults of dragon- and damselflies, serve as the second intermediate hosts (Manning and Lertprasert, 1973).

### Other miscellaneous species

Thirteen species of miscellaneous intestinal flukes having less than 10 human cases are presented in Supplementary Table. See Chai and Jung (2020) for details.

## Issues and perspectives

FBT are taxonomically diverse, and a lot of species have been involved in causing zoonotic human and animal infections. Recently, FBT infections have been included among the neglected tropical diseases by the World Health Organization. Because of the increased risk of human exposure to these parasites, the public health significance of each FBT species is expected to increase. The life cycles of many kinds of FBT have been elucidated, and a variety of human infection sources (including types of foods) have been identified. However, problems still remain including difficulties in the diagnosis of infection (fecal examination alone frequently do not provide exact/specific diagnosis) as well as poor understanding of the epidemiological situation, including the geographical distribution, prevalence, intensity of infection and other characteristics. In addition, the clinicopathological significance of each FBT infection requires further investigation.

## Supplementary material

For supplementary material accompanying this paper visit https://doi.org/10.1017/S0031182022000725. click here to view supplementary material

## References

[ref1] Abdul-Ghani RA, Loutfy N and Hassan A (2009) Myrrh and trematodes in Egypt: an overview of safety, efficacy and effectiveness profiles. Parasitology International 58, 210–214. 19446652 10.1016/j.parint.2009.04.006

[ref2] Africa CM, de Leon W and Garcia EY (1940) Visceral complications in intestinal heterophyidiasis of man. Acta Medica Philippina (Monographic Series), 1–132.

[ref3] Agatsuma T, Arakawa Y, Iwagami M, Honzako Y, Cahyangsih U, Kang SY and Hong SJ (2000) Molecular evidence of natural hybridization between *Fasciola hepatica* and *F. gigantica*. Parasitology International 49, 231–238. 11426578 10.1016/s1383-5769(00)00051-9

[ref4] Agatsuma T, Iwagami M, Sato Y, Iwashita J, Hong SJ, Kang SY, Ho LY, Su KE, Kawashima K and Abe T (2003) The origin of the triploid in *Paragonimus westermani* on the basis of variable regions in the mitochondrial DNA. Journal of Helminthology 77, 279–285. 14627442 10.1079/joh2003185

[ref5] Ahn YK and Ryang YS (1988) Epidemiological studies on *Metagonimus* infection along the Hongcheon River, Kangwon Province. Korean Journal of Parasitology 26, 207–213 (in Korean). 12811049 10.3347/kjp.1988.26.3.207

[ref6] Aka NA, Adoubryn K, Rondelaud D and Dreyfuss G (2008) Human paragonimiasis in Africa. Annals of African Medicine 7, 153–162. 19623916 10.4103/1596-3519.55660

[ref7] Ashrafi K (2010) Human dicrocoeliasis in northern Iran: two case reports from Gilan province. Annals of Tropical Medicine and Parasitology 104, 351–353. 20659397 10.1179/136485910X12647085215813

[ref8] Babbott FL Jr, Frye WWW and Gordon JE (1961) Intestinal parasites of man in Arctic Greenland. American Journal of Tropical Medicine and Hygiene 10, 185–190. 13685517 10.4269/ajtmh.1961.10.185

[ref9] Bandyopadhyay AK, Manna B and Nandy A (1989) Human infection of *Artyfechinostomum oraoni* n. sp. (Paryphostominae: Echinostomatidae) in a tribal community, ‘Oraons’ in West Bengal, India. Indian Journal of Parasitology 13, 191–196.

[ref10] Bandyopadhyay AK, Maji AK, Manna B, Bera DK, Addy M and Nandy A (1995) Pathogenicity of *Artyfechinostomum oraoni* in naturally infected pigs. Tropical Medicine and Parasitology 46, 138–139. 8525286

[ref11] Bargues MD and Mas-Coma S (2005) Reviewing lymnaeid vectors of fascioliasis by ribosomal DNA sequence analyses. Journal of Helminthology 79, 257–267. 16153320 10.1079/joh2005297

[ref12] Bayssade-Dufour C, Chermette R, Šundić D and Radujković B (2014) *Paragonimus gondwanensis* n. sp. (Digenea, Paragonimidae), parasite of mammals (humans and carnivores) in Cameroon. Ecologica Montenegrina 1, 256–267.

[ref13] Bayssade-Dufour C, Chermette R, Šundić D and Radujković B (2015) *Paragonimus kerberti* n. sp. (Digenea, Paragonimidae), a parasite of carnivores in Cameroon. Ecologica Montenegrina 2, 271–277.

[ref14] Beaver PC, Jung RC and Cupp EW (1984) Clinical Parasitology, 9th Edn. Philadelphia, USA: Lea & Febiger.

[ref15] Béland JE, Boone J, Donevan RE and Mankiewicz E (1969) Paragonimiasis (the lung fluke) report of four cases. American Review of Respiratory Disease 99, 261–271. 5767005 10.1164/arrd.1969.99.2.261

[ref16] Belizario VY, Geronilla GG, Anastacio MBM, de Leon WU, Suba-an AP, Sebastian AC and Bangs MJ (2007) *Echinostoma malayanum* infection, the Philippines. Emerging Infectious Diseases 13, 1130–1131. 18214206 10.3201/eid1307.061486 PMC2878229

[ref17] Besprozvannykh VV, Tatonova YV and Shumenko PG (2018) Life cycle, morphology of developmental stages of *Metorchis ussuriensis* sp. nov. (Trematoda: Opisthorchiidae), and phylogenetic relationships with other opisthorchiids. Journal of Zoological Systematics and Evolutionary Research 57, 24–40. 10.1111/jzs.12230

[ref18] Blair D (2019) Paragonimiasis. In Toledo R and Fried B (eds), Digenetic Trematodes. Advances in Experimental Medicine and Biology, vol. 1154. pp. 105–138. Available at 10.1007/978-3-030-18616-6_5. 31297761

[ref19] Blair D, Agatsuma T, Watanobe T, Okamoto M and Ito A (1997) Geographical genetic structure within the human lung fluke, *Paragonimus westermani*, detected from DNA sequences. Parasitology 115, 411–417. 9364568 10.1017/s0031182097001534

[ref20] Blair D, Xu ZB and Agatsuma T (1999 *a*) Paragonimiasis and the genus *Paragonimus*. Advances in Parasitology 42, 113–122. 10050273 10.1016/s0065-308x(08)60149-9

[ref21] Blair D, Wu B, Chang ZS, Gong X, Agatsuma T, Zhang YN, Chen SH, Lin JX, Chen MG, Waikagul J, Guevara AG, Feng Z and Davis GM (1999 *b*) A molecular perspective on the genera *Paragonimus* Braun, *Euparagonimus* Chen and *Pagumogonimus* Chen. Journal of Helminthology 73, 295–299. 10654398

[ref22] Blair D, Chang Z, Chen M, Cui A, Wu B, Agatsuma T, Iwagami M, Corlis D, Fu C and Zhan X (2005) *Paragponimus skrjabini* Chen, 1959 (Digenea: Paragonimidae) and related species in eastern Asia: a combined molecular and morphological approach to identification and taxonomy. Systematic Parasitology 60, 1–21. 15791397 10.1007/s11230-004-1378-5

[ref23] Blair D, Agatsuma T and Wang W (2007) Paragonimiasis. In Murrell KD and Fried B (eds), Food-borne Parasitic Zoonoses: Fish and Plant-Borne Parasites. World Class Parasites, vol. 11. New York, USA: Springer, pp. 117–150.

[ref24] Boland JM, Vaszar LT, Jones JL, Mathison BA, Rovzar MA, Colby TV, Leslie KO and Tazelaar HD (2011) Pleuropulmonary infection by *Paragonimus westermani* in the United States: a rare cause of eosinophilic pneumonia after ingestion of live crabs. American Journal of Surgical Pathology 35, 707–713. 21415702 10.1097/PAS.0b013e318211acd9

[ref25] Brenes Madrigal R, Rodriguez-Ortiz B, Vargas Solano G, Ocamp Obando EM and Ruiz Sotela PJ (1982) Cerebral hemmorhagic lesions produced by *Paragonimus mexicanus*. Report of three cases in Costa Rica. American Journal of Tropical Medicine and Hygiene 31, 522–526. 7081546 10.4269/ajtmh.1982.31.522

[ref26] Butcher AR and Grove DI (2001) Description of life-cycle stages of *Brachylaima cribbi* n. sp. (Digenea: Brachylaimidae) derived from eggs recovered from human feces in Australia. Systematic Parasitology 49, 211–221. 11466482 10.1023/a:1010616920412

[ref27] Butcher AR and Grove DI (2005) Second intermediate host land snails and definitive host animals of *Brachylaima cribbi* in southern Australia. Parasite 12, 31–37. 15828579 10.1051/parasite/2005121031

[ref28] Butcher AR, Parasuramar P, Thompson CS and Grove DI (1998) First report of the isolation of an adult worm of the genus *Brachylaima* (Digenea: Brachylaimidae), from the gastrointestinal tract of a human. International Journal for Parasitology 28, 607–610. 9602383 10.1016/s0020-7519(97)84372-x

[ref29] Butcher AR, Palethope HM and Grove DI (2003) Response to re-infection with *Brachylaima cribbi* in immunocompetent and immunodeficient mice. Parasitology International 52, 219–228. 14550477 10.1016/s1383-5769(03)00026-6

[ref30] Calvopiña M, Cevallos W, Kumazawa H and Eisenberg J (2011) High prevalence of human liver infection by *Amphimerus* spp. flukes, Ecuador. Emerging Infectious Diseases 17, 2331–2334. 22172165 10.3201/eid1712.110373 PMC3311191

[ref31] Calvopiña M, Romero D, Castañeda B, Hashiguchi Y and Sugiyama H (2014) Current status of *Paragonimus* and paragonimiasis in Ecuador. Memorias Instituto Oswaldo Cruz 109, 849–855. 10.1590/0074-0276140042 PMC4296488 25410987

[ref32] Calvopiña M, Cevallos W, Atherton R, Saunders M, Small A, Kumazawa H and Sugiyama H (2015) High prevalence of the liver fluke *Amphimerus* sp. In domestic cats and dogs in an area for human amphimeriasis in Ecuador. PLoS Neglected Tropical Diseases 9, e000526. 10.1371/journal.pntd.0003526 PMC4315407 25647171

[ref33] Calvopiña M, Romero-Alvarez D, Rendon M, Takagi H and Sugiyama H (2018) *Hypolobocera guayaquilensis* (Decapoda: Pseudothelphusidae): a new crab intermediate host of *Paragonimus mexicanus* in Manabí Province, Ecuador. Korean Journal of Parasitology 56, 189–194. 29742874 10.3347/kjp.2018.56.2.189 PMC5976022

[ref34] Cevallos W, Calvopiña M, Nipáz V, Vicente-Santiago B, López-Alban J, Fernández-Soto P, Guevara A and Muro A (2017) Enzyme-linked immunosorbent assay for diagnosis of *Amphimerus* spp. liver fluke infection in humans. Memorias Instituto Oswaldo Cruz (Rio de Janeiro) 112, 364–369. 10.1590/0074-02760160426 PMC5398163 28443982

[ref35] Chai JY (2007) Intestinal flukes. In Murrell KD and Fried B (eds), Food-borne Parasitic Zoonoses: Fish and Plant-borne Parasites. World Class Parasites, vol. 11. New York, USA: Springer, pp. 53–115.

[ref36] Chai JY (2009) Echinostomes in humans. In Fried B and Toledo R (eds), The Biology of Echinostomes. New York, USA: Springer, pp. 147–183.

[ref37] Chai JY (2013 *a*) Praziquantel treatment in trematode and cestode infections: an update. Infection and Chemotherapy 45, 32–43. 24265948 10.3947/ic.2013.45.1.32 PMC3780935

[ref38] Chai JY (2013 *b*) Paragonimiasis. In Garcia HH, Tanowitz HB and Del Brutto OH (eds), Neuroparasitology and Tropical Neurology. Handbook of Clinical Neurology. Vol. 114 (3rd Series). Amsterdam, The Netherlands: Elsevier, pp. 283–296.

[ref39] Chai JY (2019) Human Intestinal Flukes. From Discovery to Treatment and Control. Dordrecht, The Netherlands: Springer Nature B.V.

[ref40] Chai JY and Jung BK (2017) Fishborne zoonotic heterophyid infections: an update. Food and Waterborne Parasitology 8–9, 33–63. 10.1016/j.fawpar.2017.09.001 PMC7034020 32095640

[ref41] Chai JY and Jung BK (2018) *Paragonimus* spp. In Robertson L (ed.), Global Water Pathogen Project. Part 3. Specific Excreted Pathogens: Environmental and Epidemiological Aspects. Section IV. Helminths. Michigan State University, E. Lansing, MI, USA: UNESCO, pp. 1–18.

[ref42] Chai JY and Jung BK (2019) Epidemiology of trematode infections: an update. In Toledo R and Fried B (eds), Digenetic Trematodes. Advances in Experimental Medicine and Biology, vol. 1154. pp. 359–409. Available at 10.1007/978-3-030-18616-6_12. 31297768

[ref43] Chai JY and Jung BK (2020) Foodborne intestinal flukes: a brief review of epidemiology and geographical distribution. Acta Tropica 201, 105210. 31600520 10.1016/j.actatropica.2019.105210

[ref44] Chai JY and Lee SH (2002) Food-borne intestinal trematode infections in the Republic of Korea. Parasitology International 51, 129–154. 12113752 10.1016/s1383-5769(02)00008-9

[ref45] Chai JY, Hong SJ, Son DW, Lee SH and Seo BS (1985) Metacercariae of the *Echinochasmus japonicus* encysted in a fresh water fish, *Pseudorasbora parva*, and their development in experimental mice. Korean Journal of Parasitology 23, 221–229. 12888664 10.3347/kjp.1985.23.2.221

[ref46] Chai JY, Hong SJ, Lee SH and Seo BS (1988) *Stictodora* sp. (Trematoda: Heterophyidae) recovered from a man in Korea. Korean Journal of Parasitology 26, 127–132. 12811059 10.3347/kjp.1988.26.2.127

[ref47] Chai JY, Huh S, Yu JR, Kook J, Jung KC, Park EC, Sohn WM, Hong ST and Lee SH (1993) An epidemiological study of metagonimiasis along the upper reaches of the Namhan River. Korean Journal of Parasitology 31, 99–108. 8343462 10.3347/kjp.1993.31.2.99

[ref48] Chai JY, Han ET, Park YK, Guk SM and Lee SH (2001) *Acanthoparyphium tyosenense*: the discovery of human infection and identification of its source. Journal of Parasitology 87, 794–800. 11534643 10.1645/0022-3395(2001)087[0794:ATTDOH]2.0.CO;2

[ref49] Chai JY, Choi MH, Yu JR and Lee SH (2003) *Gymnophalloides seoi*: a new human intestinal trematode. Trends in Parasitology 19, 109–112. 12643990 10.1016/s1471-4922(02)00068-5

[ref50] Chai JY, Park JH, Han ET, Shin EH, Kim JL, Guk SM, Hong KS, Lee SH and Rim HJ (2004) Prevalence of *Heterophyes nocens* and *Pygidiopsis summa* infections among residents of the western and southern coastal islands of the Republic of Korea. American Journal of Tropical Medicine and Hygiene 71, 617–622. 15569794

[ref51] Chai JY, Murrell KD and Lymbery AJ (2005) Fish-borne parasitic zoonoses: status and issues. International Journal for Parasitology 35, 1233–1254. 16143336 10.1016/j.ijpara.2005.07.013

[ref52] Chai JY, Han ET, Guk SM, Shin EH, Sohn WM, Yong TS, Eom KS, Lee KH, Jeong HG, Ryang YS, Hoang EH, Phommasack B, Insisiengmay B, Lee SH and Rim HJ (2007) High prevalence of liver and intestinal fluke infections among residents of Savannakhet Province, Laos. Korean Journal of Parasitology 45, 213–218. 17876167 10.3347/kjp.2007.45.3.213 PMC2526321

[ref53] Chai JY, Shin EH, Lee SH and Rim HJ (2009) Foodborne intestinal flukes in Southeast Asia. Korean Journal of Parasitology 47, S69–S102. 19885337 10.3347/kjp.2009.47.S.S69 PMC2769220

[ref54] Chai JY, Sohn WM, Na BK and Nguyen VD (2011) *Echinostoma revolutum*: metacercariae in *Filopaludina* snails from Nam Dihn Province, Vietnam, and adults from experimental hamsters. Korean Journal of Parasitology 49, 449–455. 22355218 10.3347/kjp.2011.49.4.449 PMC3279689

[ref55] Chai JY, De NV and Sohn WM (2012 *a*) Foodborne trematode metacercariae in fish from northern Vietnam and their adults recovered from experimental hamsters. Korean Journal of Parasitology 50, 317–325. 23230329 10.3347/kjp.2012.50.4.317 PMC3514423

[ref56] Chai JY, Sohn WM, Yong TS, Eom KS, Min DY, Hoang EH, Phammasack B, Insisiengmay B and Rim HJ (2012 *b*) Echinostome flukes recovered from humans in Khammouane Province, Lao PDR. Korean Journal of Parasitology 50, 269–272. 22949759 10.3347/kjp.2012.50.3.269 PMC3428577

[ref57] Chai JY, Yong TS, Eom KS, Min DY, Jeon HK, Kim TY, Jung BK, Sisabath L, Insisiengmay B, Phommasack B and Rim HJ (2013 *a*) Hyperendemicity of *Haplorchis taichui* infection among riparian people in Saravane and Champasak Province, Lao PDR. Korean Journal of Parasitology 51, 305–311. 23864741 10.3347/kjp.2013.51.3.305 PMC3712104

[ref58] Chai JY, Sohn WM, Yong TS, Eom KS, Min DY, Lee MY, Lim H, Insisiengmay B, Phommasack B and Rim HJ (2013 *b*) *Centrocestus formosanus* (Heterophyidae): human infections and the infection source in Lao PDR. Journal of Parasitology 99, 531–536. 23116489 10.1645/12-37.1

[ref59] Chai JY, Sohn WM, Na BK, Yong TS, Eom KS, Yoon CH, Hoang EH, Jeoung HG and Socheat D (2014) Zoonotic trematode metacercariae in fish from Phnom Penh and Pursat, Cambodia. Korean Journal of Parasitology 52, 35–40. 24623879 10.3347/kjp.2014.52.1.35 PMC3948991

[ref60] Chai JY, Sohn WM, Jung BK, Yong TS, Eom KS, Min DY, Insisiengmay B, Insisiengmay S, Phommasack B and Rim HJ (2015) Intestinal helminths recovered from humans in Xieng Khouang Province, Lao PDR with a particular note on *Haplorchis pumilio* infection. Korean Journal of Parasitology 53, 439–445. 26323842 10.3347/kjp.2015.53.4.439 PMC4566498

[ref61] Chai JY, Sohn WM, Na BK, Jeoung HG, Sinuon M and Socheat D (2016) *Stellantchasmus falcatus* (Digenea: heterophyidae) in Cambodia: discovery of metacercariae in mullets and recovery of adult flukes in an experimental hamster. Korean Journal of Parasitology 54, 537–541. 27658608 10.3347/kjp.2016.54.4.537 PMC5040078

[ref62] Chai JY, Sohn WM, Cho J, Eom KS, Yong TS, Min DY, Hoang EH, Phommasack B, Insisiengmay B and Rim HJ (2018) *Echinostoma ilocanum* infection in two residents of Savannakhet Province, Lao PDR. Korean Journal of Parasitology 56, 77–81. 10.3347/kjp.2018.56.1.75 PMC5858667 29529854

[ref63] Chai JY, Lee SH, Rim HJ, Sohn WM and Phommasack B (2019 *a*) Infection status with zoonotic trematode metacercariae in fish from Lao PDR. Acta Tropica 199, 105100. 31404522 10.1016/j.actatropica.2019.105100

[ref64] Chai JY, Chang T, Jung BK, Shin H, Soh WM, Eom KS, Yong TS, Min DY, Phommasack B, Insisiengmay B and Rim HJ (2019 *b*) *Echinostoma caninus* n. comb. (Trematoda: Echinostomatidae) infection in eleven riparian people in Khammouane Province, Lao PDR. Korean Journal of Parasitology 57, 451–456. 31533415 10.3347/kjp.2019.57.4.451 PMC6753300

[ref65] Chai JY, Cho J, Chang T, Jung BK and Sohn WM (2020) Taxonomy of *Echinostoma revolutum* and 37-collar-spined Echinostoma spp.: a historical review. Korean Journal of Parasitology 58, 343–371. 32871630 10.3347/kjp.2020.58.4.343 PMC7462802

[ref66] Chai JY, Jung BK and Hong SJ (2021 *a*) Albendazole and mebendazole as anti-parasitic and anti-cancer agents: an update. Korean Journal of Parasitology 59, 189–225. 34218593 10.3347/kjp.2021.59.3.189 PMC8255490

[ref67] Chai JY, Sohn WM, Cho J, Jung BK, Chang T, Lee KH, Khieu V and Huy R (2021 *b*) *Echinostoma mekongi*: discovery of its metacercarial stage in snails, *Filopaludina martensi cambodjiensis*, in Pursat Province, Cambodia. Korean Journal of Parasitology 59, 47–53. 33684986 10.3347/kjp.2021.59.1.47 PMC7939970

[ref68] Chantima K, Chai JY and Wongsawad C (2013) *Echinostoma revolutum*: freshwater snails as the second intermediate hosts in Chiang Mai, Thailand. Korean Journal of Parasitology 51, 183–189. 23710085 10.3347/kjp.2013.51.2.183 PMC3662061

[ref69] Chen M and Mott KE (1990) Progress in assessment of morbidity due to *Fasciola hepatica* infection: a review of recent literature. Tropical Diseases Bulletin 87, R1–R38.

[ref70] Chen M, Lu Y, Hua X and Mott KE (1994) Progress in assessment of morbidity due to *Clonorchis sinensis* infection: a review of recent literature. Tropical Diseases Bulletin 91, R7–65.

[ref71] Cho J, Jung BK, Chang T, Sohn WM, Sinuon M and Chai JY (2020) *Echinostoma mekongi* n. sp. (Digenea: Echinostomatidae) from riparian people along the Mekong River in Cambodia. Korean Journal of Parasitology 58, 431–443. 32871637 10.3347/kjp.2020.58.4.431 PMC7462798

[ref72] Choi DW (1990) *Paragonimus* and paragonimiasis in Korea. Korean Journal of Parasitology 28(suppl.), 79–102. 10.3347/kjp.1990.28.suppl.79 2133425

[ref73] Choi MH, Kim SH, Chung JH, Jang HJ, Eom JH, Chung BS, Sohn WM, Chai JY and Hong ST (2006) Morphological observations of *Echinochasmus japonicus* cercariae and the in vitro maintenance of its life cycle from cercariae to adults. Journal of Parasitology 92, 236–241. 16729678 10.1645/GE-354R1.1

[ref74] Choo JD, Suh BS, Lee HS, Lee JS, Song CJ, Shin DW and Lee YH (2003) Chronic cerebral paragonimiasis combined with aneurysmal subarachnoid hemorrhage. American Journal of Tropical Medicine and Hygiene 69, 466–469. 14695081

[ref75] Chung OS, Lee HJ, Kim YM, Sohn WM, Kwak SJ and Seo M (2011) First report of human infection with *Gynaecotyla squatarolae* and first Korean record of *Haplorchis pumilio* in a patient. Parasitology International 60, 227–229. 21081175 10.1016/j.parint.2010.11.003

[ref76] Coogle B, Sosland S and Bahr NC (2021) A clinical review of human disease due to *Paragonimus kellicotti* in North America. Parasitology 148, 1–7. 10.1017/s0031182021001359 PMC9415338 35965058

[ref77] Cumberlidge N, Rollinson D, Vercruysse J, Tchuem Tchuenté LA, Webster B and Clark PF (2018) *Paragonimus* and paragonimiasis in West and Central Africa: unsolved questions. Parasitology 145, 1748–1757. 10.1017/S0031192018001439 30210013

[ref78] Dar JS, Shabir U, Dar SA and Ganai BA (2020) Molecular characterization and immunodiagnostics of *Dicrocoelium dendriticum* species isolated from sheep of north-west Himalayan region. Journal of Helminthology 94, e174. 32684172 10.1017/S0022149X20000565

[ref79] David AR (1997) Disease in Egyptian mummies: the contribution of new technologies. Lancet (London, England) 349, 1760–1763. 9193394 10.1016/s0140-6736(96)10221-x

[ref80] Devi KR, Narain K, Mahanta J, Nirmolia T, Blair D, Saikia SP and Agatsuma T (2013) Presence of three distinct genotypes within the *Paragonimus westermani* complex in northern India. Parasitology 140, 76–86. 22917216 10.1017/S0031182012001229

[ref81] Doanh PN, Shinohara A, Horii Y, Habe S, Nawa Y and Le NT (2007) Description of a new lung fluke species, *Paragonimus vietnamensis* sp. nov. (Trematoda: Paragonimidae), found in northern Vietnam. Parasitology Research 101, 1495–1501. 17674049 10.1007/s00436-007-0666-9

[ref82] Doanh PN, Shinohara A, Horii Y, Habe S and Nawa Y (2009) Discovery of *Paragonimus westermani* in Vietnam and its molecular phylogenetic status in *P. westermani* complex. Parasitology Research 104, 1149–1159. 19083013 10.1007/s00436-008-1302-z

[ref83] Doanh PN, Horii Y and Nawa Y (2013 *a*) *Paragonimus* and paragonimiasis in Vietnam: an update. Korean Journal of Parasitology 51, 621–627. 24516264 10.3347/kjp.2013.51.6.621 PMC3916448

[ref84] Doanh PN, Hien HV, Nonaka N, Horii Y and Nawa Y (2013 *b*) Discovery of *Paragonimus skrjabini* in Vietnam and its phylogenetic status in the *Paragonimus skrjabini* complex. Journal of Helminthology 87, 450–460. 23067567 10.1017/S0022149X1200065X

[ref85] Doanh PN, Thaenkham U, An PT, Hien HV, Horii Y and Nawa Y (2015) Metacercarial polymorphism and genetic variation of *Paragonimus heterotremus* (Digenea: Paragonimidae), and a re-appraisal of the taxonomic status of *Paragonimus pseudoheterotremus*. Journal of Helminthology 89, 182–188. 24229574 10.1017/S0022149X13000734

[ref86] Drabick JJ, Egan JE, Brown SL, Vick RG, Sandman BM and Neafie RC (1988) Dicrocoeliasis (lancet fluke disease) in an HIV seropositive man. Journal of the American Medical Association 259, 567–568. 3336179

[ref87] Duflot M, Setbon T, Midelet G, Brauge T and Gay M (2021) A review of molecular identification tools for the Opisthorchioidea. Journal of Molecular Methods 187, 106258. 10.1016/j.mimet.2021.106258 34082051

[ref88] Eastburn RL, Tritsche TR and Terhune CA Jr (1987) Human intestinal infection with *Nanophyetus salmincola* from salmonid fishes. American Journal of Tropical Medicine and Hygiene 36, 586–591. 3578655 10.4269/ajtmh.1987.36.586

[ref89] Fairweather I (2009) Triclabendazole progress report, 2005–2009: an advancement of learning? Journal of Helminthology 83, 139–150. 19366485 10.1017/S0022149X09321173

[ref90] Fedorov KP, Naumov VA, Kuznetsova VG and Belov GF (2002) [Some real problems of human opisthorchiasis]. Meditsinskaia Parazitologiia i Parazitarnye Bolezni 3, 7–9. 12298176

[ref91] Fedorova OS, Fedotova MM, Sokolova TS, Golovach EA, Kovshirina YV, Ageeva TS, Kovshirina AE, Kobyakova OS, Ogorodova LM and Odermatt P (2018) *Opisthorchis felineus* infection prevalence in Western Siberia: a review of Russian literature. Acta Tropica 178, 196–204. 29191519 10.1016/j.actatropica.2017.11.018

[ref92] Fischer PU, Curtis KC, Marcos LA and Weil GJ (2011) Molecular characterization of the North American lung fluke *Paragonimus kellicotti* in Missouri and its development in Mongolian gerbils. American Journal of Tropical Medicine and Hygiene 84, 1005–1011. 21633042 10.4269/ajtmh.2011.11-0027 PMC3110363

[ref93] Flavell DJ (1981) Liver-fluke infection as an aetiological factor in bile-duct carcinoma of man. Transactions of the Royal Society of Tropical Medicine and Hygiene 75, 814–824. 6277052 10.1016/0035-9203(81)90419-3

[ref94] Friant S, Brown K, Saari MT, Segel NH, Slezak J and Goldberg TL (2015) Lung fluke (*Paragonimus africanus*) infects Nigerian red-capped mangabeys and causes respiratory disease. International Journal for Parasitology: Parasites and Wildlife 4, 329–332. 26543803 10.1016/j.ijppaw.2015.08.003 PMC4564387

[ref95] Fried B and Abruzzi A (2010) Food-borne trematode infections of humans in the United States of America. Parasitology Research 106, 1263–1280. 20352454 10.1007/s00436-010-1807-0

[ref96] Fürst T, Keiser J and Utzinger J (2012 *a*) Global burden of human food-borne trematodiasis: a systematic review and meta-analysis. Lancet Infectious Diseases 12, 210–221. 22108757 10.1016/S1473-3099(11)70294-8

[ref97] Fürst T, Sayasone S, Odermatt P, Keiser J and Utzinger J (2012 *b*) Manifestation, diagnosis, and management of food-borne trematodiasis. British Medical Journal 344, e4093. 22736467 10.1136/bmj.e4093

[ref98] Fürst T, Yongvanit P, Khuntikeo N, Lun ZR, Haagsma JA, Torgerson PR, Odermatt P, Bürli C, Chitnis N and Sithithaworn P (2019) Food-borne trematodiases in East Asia: epidemiology and burden. In Utzinger Z, Yap P, Bratschi M and Steinmann P (eds), Neglected Tropical Diseases-East Asia, Neglected Tropical Diseases. Switzerland AG: Springer Cham, pp. 13–38. 10.1007/978-3-030-12008-5_2

[ref99] Garcia LS (2016) Diagnostic Medical Parasitology, 6th Edn. Washington, DC, USA: ASM Press.

[ref100] Guk SM, Shin EH, Kim JL, Sohn WM, Hong KS, Yoon CH, Lee SH, Rim HJ and Chai JY (2007) A survey of *Heterophyes nocens* and *Pygidiopsis summa* metacercariae in mullets and gobies along the coastal areas of the Republic of Korea. Korean Journal of Parasitology 45, 205–211. 17876166 10.3347/kjp.2007.45.3.205 PMC2526326

[ref101] Haseeb AN, El Shazly AM, Arafa MAS and Morsy AT (2002) A review on fascioliasis in Egypt. Journal of the Egyptian Society of Parasitology 32, 317–335. 12049266

[ref102] Hayashi K, Tang WQ, Ohari Y, Ohtori M, Mohanta UK, Matsuo K, Sato H and Itagaki T (2017) Phylogenetic relationship between *Dicrocoelium chinensis* populations in Japan and China based on mitochondrial *nad*1 gene sequences. Parasitology Research 116, 2605–2609. 28735469 10.1007/s00436-017-5557-0

[ref103] Hernández-Chea R, Jiménez-Rocha AE, Castro R, Blair D and Dolz G (2017) Morphological and molecular characterization of the metacercaria of *Paragonimus caliensis*, as a separate species from *P. mexicanus* in Costa Rica. Parasitology International 66, 126–133. 28027969 10.1016/j.parint.2016.12.006

[ref104] Hong ST and Fang Y (2012) *Clonorchis sinensis* and clonorchiasis, an update. Parasitology International 61, 17–24. 21741496 10.1016/j.parint.2011.06.007

[ref105] Hong ST, Cho TK, Hong SJ, Chai JY, Lee SH and Seo BS (1984) Fifteen human cases of *Fibricola seoulensis* infection in Korea. Korean Journal of Parasitology 22, 61–65. 12891032 10.3347/kjp.1984.22.1.61

[ref106] Hotez P and Alibek K (2011) Central Asia's hidden burden of neglected tropical diseases. PLoS Neglected Tropical Diseases 5, e1224. 21980541 10.1371/journal.pntd.0001224 PMC3181239

[ref107] Hou PC (1956) The relationship between primary carcinoma of the liver and infestation with *Clonorchis sinensis*. Journal of Pathology and Bacteriology 72, 239–246. 13368000 10.1002/path.1700720130

[ref108] Huang WY, He B, Wang CR and Zhu XQ (2004) Characterisation of *Fasciola* species from Mainland China by ITS2 ribosomal DNA sequence. Veterinary Parasitology 120, 75–83. 15019145 10.1016/j.vetpar.2003.12.006

[ref109] Im JG, Kong Y, Shin YM, Yang SO, Song JG, Han MC, Kim CW, Cho SY and Ham EK (1993) Pulmonary paragonimiasis: clinical and experimental studies. Radiographics 13, 575–586. 8316665 10.1148/radiographics.13.3.8316665

[ref110] Itagaki T, Kikawa M, Sakaguchi K, Shimo J, Terasaki K, Shibahara T and Fukuda F (2005) Genetic characterization of parthenogenic *Fasciola* sp. in Japan on the basis of the sequences of ribosomal and mitochondrial DNA. Parasitology 131, 679–685. 16255826 10.1017/S0031182005008292

[ref111] Ito J (1964) *Metagonimus* and other human heterophyid trematodes. Progress of Medical Parasitology in Japan 1, 314–393.

[ref112] Iwagami M, Monroy C, Rosas MA, Pinto MR, Guevara AG, Vieira JC, Agatsuma Y and Agatsuma T (2003) A molecular phylogeographic study based on DNA sequences from individual metacercariae of *Paragonimus mexicanus* from Guatemala and Ecuador. Journal of Helminthology 77, 33–38. 12590662 10.1079/JOH2002147

[ref113] Iwagami M, Rajapakse RPVJ, Paranagama W, Okada T, Kano S and Agatsuma T (2008) Ancient divergence of *Paragonimus westermani* in Sri Lanka. Parasitology Research 102, 845–852. 18193283 10.1007/s00436-007-0820-4

[ref114] Kamo H, Nishida H, Hatsushika R and Tomimura T (1961) On the occurrence of a new lung fluke, *Paragonimus miyazakii* n. sp. in Japan (Trematoda: Troglotrematidae). Yonago Acta Medica 5, 43–52 (in Japanese).

[ref115] Kanev I (1994) Life-cycle, delimitation and redescription of *Echinostoma revolutum* (Froelich, 1982) (Trematoda: Echinostomatidae). Systematic Parasitology 28, 125–144.

[ref116] Kang BK, Jung BK, Lee YS, Hwang IK, Lim H, Cho J, Hwang JH and Chai JY (2014) A case of *Fasciola hepatica* infection mimicking cholangiocarcinoma and ITS-1 sequencing of the worm. Korean Journal of Parasitology 52, 193–196. 24850964 10.3347/kjp.2014.52.2.193 PMC4028458

[ref117] Kannagara DWW and Karunaratne GMS (1969) *Paragonimus siamensis*: the fourth lung fluke reported from Ceylon. Ceylon Journal of Medical Science 18, 61–65.

[ref118] Keiser J, Engels D, Büscher G and Utzinger J (2005) Triclabendazole for the treatment of fascioliasis and paragonimiasis. Expert Opinion on Investigational Drugs 14, 1513–1526. 16307491 10.1517/13543784.14.12.1513

[ref119] Khamidullin RI, Liubina VS, Khamidullin IR and Medinskiĭ BL (1991) [Trematodiases in Tartaria]. Meditsinskaia Parazitologiia I Parazitarnye Bolezi 2, 60–61. 1829788

[ref120] Khamidullin RI, Fomina OA, Sultanaeva EG and Khamidullin IR (1995) [Opisthorchiasis and pseudamphistomiasis on the territory of the middle Volga valley]. Meditsinskaia Parazitologiia I Parazitarnye Bolezi 1, 40–42. 7770020

[ref121] Khan MA, Afshan K, Nazar M, Firasat S, Chaudhry U and Sargison ND (2021) Molecular confirmation of *Dicrocoelium dendriticum* in the Himalayan ranges of Pakistan. Parasitology International 81, 102276. 33370606 10.1016/j.parint.2020.102276

[ref122] Khandelwal N, Shaw J and Jain MK (2008) Biliary parasites: diagnostic and therapeutic strategies. Current Treatment Opinions in Gastroenterology 11, 85–95. 10.1007/s11938-008-0020-z 18321435

[ref123] Khieu V, Fürst T, Miyamoto K, Yong TS, Chai JY, Huy R, Muth S and Odermatt P (2019) Is *Opisthorchis viverrini* emerging in Cambodia? Advances in Parasitology 103, 31–73. 30878058 10.1016/bs.apar.2019.02.002

[ref124] Kim DC (1984 *a*) *Paragonimus westermani*: life cycle, intermediate hosts, transmission to man and geographical distribution in Korea. Arzneimittel-Forschung/Drug Research 34, 1180–1183. 6542389

[ref125] Kim YI (1984 *b*) Liver carcinoma and liver fluke infection. Arzneimittel-Forschung/Drug Research 34, 1121–1126. 6095876

[ref126] Kim YK, Yu JE, Chung EY and Chung PR (2004) *Acanthoparyphium tyosenense* (Digenea: Echinostomatidae): experimental confirmation of the cercaria and its complete life history in Korea. Journal of Parasitology 90, 97–102. 15040673 10.1645/GE-3145

[ref127] Kim MJ, Kim SH, Lee SO, Choi SH, Kim YS, Woo JH, Yoon YS, Kim KW, Cho J, Chai JY and Chong YP (2017) A case of ectopic peritoneal paragonimiasis mimicking diverticulitis or abdominal abscess. Korean Journal of Parasitology 55, 313–317. 28719956 10.3347/kjp.2017.55.3.313 PMC5523897

[ref128] Kim JY, Yong TS, Rim HJ, Chai JY, Min DY, Eom KS, m Sohn WM, Lim JH, Choi D, Insisiengmay S, Phommasack B and Insisiengmay B (2018) Ultrasonographic investigation of cholangiocarcinoma in Lao PDR. Acta Tropica 182, 128–134. 29486176 10.1016/j.actatropica.2018.02.031

[ref129] King EVJ (1971) Human infection with *Dicrocoelium hospes* in Sierra Leone. Journal of Parasitology 57, 989. 5133904

[ref130] Kino H, Oishi H, Ohno Y and Mitsuru I (2002) An endemic human infection with *Heterophyes nocens* Onji et Nishio, 1916 at Mikkabi-cho, Shizuoka, Japan. Japanese Journal of Tropical Medicine and Hygiene 30, 301–304.

[ref131] Kino H, Suzuki T, Oishi H, Suzuki S, Yamagiwa S and Ishiguro M (2006) Geographical distribution of *Metagonimus yokogawai* and *M. miyatai* in Shizuoka Prefecture, Japan, and their site preferences in the sweetfish, *Plecoglossus altivelis*, and hamsters. Parasitology International 55, 201–206. 16807078 10.1016/j.parint.2006.05.001

[ref132] Koh EJ, Kim SK, Wang KC, Chai JY, Chong S, Park SH, Cheon JE and Phi JH (2012) The return of an old worm: cerebral paragonimiasis presenting with intracerebral hemorrhage. Journal of the Korean Medical Science 27, 1428–1432. 10.3346/jkms.2012.27.11.1428 PMC3492682 23166429

[ref133] Korea Association of Health Promotion (2013) Statistics on the Prevalence of Intestinal Parasitic Infections in Korea – The 8th Report (Monographic Series in Korean). Seoul, Korea: Korean CDC and Korea Association of Health Promotion.

[ref134] Kumchoo K, Wongsawad C, Vanittanakom P, Chai JY and Rojanapaibul A (2007) Effect of niclosamide on the tegumental surface of *Haplorchis taichui* using scanning electron microscopy. Journal of Helminthology 81, 329–337. 17588285 10.1017/S0022149X07381108

[ref135] Kuntz RE, Lawless DK, Langbehn HR and Malakatis GM (1958) Intestinal protozoa and helminths in the peoples of Egypt living in different type localities. American Journal of Tropical Medicine and Hygiene 7, 630–639. 13595208 10.4269/ajtmh.1958.7.630

[ref136] Kusner DR and King CH (1993) Cerebral paragonimiasis. Seminar in Neurology 13, 201–208. 10.1055/s-2008-1041126 8356355

[ref137] Kuznetsova VG, Naumov VA and Belov GF (2000) Metorchiasis in the residents of Novosibirsk area, Russia. Cytobios 102, 33–34. 10822796

[ref138] Kyung SY, Cho YK, Kim YJ, Park JW, Jeong SH, Lee JI, Sung YM and Lee SP (2011) A paragonimiasis patient with allergic reaction to praziquantel and resistant to triclabendazole: successful treatment after desensitization to praziquantel. Korean Journal of Parasitology 49, 73–77. 21461273 10.3347/kjp.2011.49.1.73 PMC3063930

[ref139] Lai DH, Hong XK, Su BX, Liang C, Hide G, Zhang X, Yu X and Lun ZR (2016) Current status of *Clonorchis sinensis* and clonorchiasis in China. Transactions of the Royal Society of Tropical Medicine and Hygiene 110, 21–27. 26740359 10.1093/trstmh/trv100

[ref140] Landaverde-González P, Osgood J, Quinoñez CAM, Monzón V, Rodas A and Monroy C (2022) The effect of landscape and human settlement on the genetic differentiation of *Paragonimus* species in Mesoamerica. International Journal for Parasitology 52, 13–21. 34371019 10.1016/j.ijpara.2021.05.010

[ref141] Lane MA, Barsanti MC, Santos CA, Young M, Lubner SJ and Weil GJ (2009) Human paragonimiasis in North America following ingestion of raw crayfish. Clinical Infectious Diseases 49, e55–e61. 19681705 10.1086/605534

[ref142] Lane MA, Marcos LA, Onen NF, Demertzis LM, Hayes EV, Davilla SZ, Nurutdinova DR, Bailey TC and Weil GJ (2012) *Paragonimus kellicotti* fluke infections in Missouri, USA. Emerging Infectious Diseases 18, 1263–1267. 22840191 10.3201/eid1808.120335 PMC3414046

[ref143] Lee SH, Hwang SW, Chai JY and Seo BS (1984) Comparative morphology of eggs of heterophyids and *Clonorchis sinensis* causing human infections in Korea. Korean Journal of Parasitology 22, 171–180. 12891010 10.3347/kjp.1984.22.2.171

[ref144] Lee SH, Yoo BH, Hong ST, Chai JY, Seo BS and Chi JG (1985) A histopathological study on the intestine of mice and rats experimentally infected by *Fibricola seoulensis*. Korean Journal of Parasitology 23, 58–72. 12888687 10.3347/kjp.1985.23.1.58

[ref145] Lee SH, Chai JY, Yang EC, Yun CK, Hong ST and Lee JB (1988) Observation of liver pathology after praziquantel treatment in experimental *Clonorchis sinensis* infection in guinea pigs. Seoul Journal of Medicine 29, 253–262.

[ref146] Lee SH, Chai JY and Hong ST (1993) *Gymnophalloides seoi* n. sp. (Digenea: Gymnophallidae), the first report of human infection by a gymnophallid. Journal of Parasitology 79, 677–680. 8410538

[ref147] Lee SH, Kim MN, Back BY, Chai JY, Kim TH and Hwang YS (2003) Analysis of parasite-specific antibody positive patients for *Clonorchis sinensis*, *Paragonimus westermani*, cysticercus and sparganum using ELISA. Korean Journal of Laboratory Medicine 23, 126–131 (in Korean).

[ref148] Lin J, Chen Y and Li Y (2001) The discovery of natural infection of human with *Metorchis orientalis* and the investigation of its focus. Chinese Journal of Zoonoses 17, 38–53 (in Chinese).

[ref149] Liu D and Zhu XQ (2013) Fasciola. In Liu D (ed.), Molecular Detection of Human Parasitic Pathogens. Boca Raton, London, New York: CRC Press Taylor & Francis Group, pp. 343–351.

[ref150] López-Caballero J, Oceguera-Figueroa A and León-Règagnon V (2013) Detection of multiple species of human *Paragonimus* from Mexico using morphological data and molecular barcodes. Molecular Ecology Resources 13, 1–12. 23530893 10.1111/1755-0998.12093

[ref151] Lovis L, Mak TK, Phongluxa K, Soukhathammavong P, Sayasone S, Akkhavong K, Odermatt P, Keiser J and Felger I (2009) PCR diagnosis of *Opisthorchis viverrini* and *Haplorchis taichui* infections in a Lao community in an area of endemicity and comparison of diagnostic methods for parasitological field surveys. Journal of Clinical Microbiology 47, 1517–1523. 19279176 10.1128/JCM.02011-08 PMC2681877

[ref152] MacLean JD, Arthur JR, Ward BJ, Gyorkos TW, Curtis MA and Kokoskin E (1996) Common-source outbreak of acute infection due to the North American liver fluke *Metorchis conjunctus*. Lancet (London, England) 347, 154–158. 8544550 10.1016/s0140-6736(96)90342-6

[ref153] Magi B, Frati E, Bernini L, Sansoni A and Zanelli C (2009) *Dicrocoelium dendriticum*: a true infection? Le Infezioni in Medicina 2, 115–116. 19602926

[ref154] Mairiang E and Mairiang P (2003) Clinical manifestation of opisthorchiasis and treatment. Acta Tropica 88, 221–227. 14611876 10.1016/j.actatropica.2003.03.001

[ref155] Maksimova GA, Pakharukova MY, Kashina EV, Zhukova NA, Kovner AV, Lvova MN, Katokhin AV, Tolstikova TG, Sripa B and Mordvinov VA (2017) Effect of *Opisthorchis felineus* infection and dimethylnitrosamine administration on the induction of cholangiocarcinoma in Syrian hamsters. Parasitology International 66, 458–463. 26453019 10.1016/j.parint.2015.10.002 PMC4956575

[ref156] Manga-González MY and Ferreras MC (2014) Dicrocoelidae family: major species causing veterinary diseases. In Toledo R and Fried B (eds), Digenetic Trematodes. Advances in Experimental Medicine and Biology, vol. 766. pp. 393–428. Available at 10.1007/978-1-4939-0915-5_12. 24903372

[ref157] Manning GS and Lertprasert P (1973) Studies on the life cycle of *Phaneropsolus bonnei* and *Prosthodendrium molenkampi* in Thailand. Annals of Tropical Medicine and Parasitology 67, 361–365. 4202641 10.1080/00034983.1973.11686899

[ref158] Manning GS, Diggs CL, Viyanant V, Lertprasert P and Watanasirmkit K (1970) Preliminary report on *Phaneropsolus bonnei* Lie Kian Joe, 1951. A newly discovered human intestinal fluke from northeastern Thailand. Journal of the Medical Association of Thailand 53, 173–178. 5423049

[ref159] Mas-Coma S and Bargues MD (1997) Human liver flukes: a review. Research and Reviews in Parasitology (Revista Iberica de Parasitologia) 57, 145–218.

[ref160] Mas-Coma S, Bargues MD and Valero MA (2005) Fascioliasis and other plant-borne trematode zoonoses. International Journal for Parasitology 35, 1255–1278. 16150452 10.1016/j.ijpara.2005.07.010

[ref161] Mas-Coma S, Valero MA and Bargues MD (2009) *Fasciola*, lymnaeids and human fascioliasis, with a global overview on disease transmission, epidemiology, evolutionary genetics, molecular epidemiology and control. Advances in Parasitology 69, 41–146. 19622408 10.1016/S0065-308X(09)69002-3

[ref162] Mas-Coma S, Bargues MD and Valero MA (2018) Human fascioliasis infection sources, their diversity, incidence factors, analytical methods and prevention measures. Parasitology 145, 1665–1669. 29991363 10.1017/S0031182018000914

[ref163] Mas-Coma S, Valero MA and Bargues MD (2019) Fascioliasis. In Toledo R and Fried B (eds), Digenetic Trematodes. Advances in Experimental Medicine and Biology, vol. 1154. pp. 71–103. Available at 10.1007/978-3-030-18616-6_4. 31297760

[ref164] McNulty SN, Fischer PU, Townsend RR, Curtis KC, Weil GJ and Mitreva M (2014) Systems biology studies of adult *Paragonimus* lung flukes facilitate the identification of immunodominant parasite antigens. PLoS Neglected Tropical Diseases 8, e3242. 25329661 10.1371/journal.pntd.0003242 PMC4199545

[ref165] Meyers WM and Neafie RN (1976) Paragonimiasis. In Binford CH and Conner DH (eds), Pathology of Tropical and Extraordinary Diseases, vol. 2. Washington, DC, USA: Armed Forces Institute of Pathology, pp. 517–523.

[ref166] Miyazaki I (1991) An Illustrated Book of Helminthic Zoonoses. Tokyo, Japan: International Medical Foundation of Japan, pp. 76–146.

[ref167] Miyazaki I and Habe S (1976) A newly recognized mode of human infection with lung flukes, *Paragonimus westermani* (Kerbert, 1878). Journal of Parasitology 48, 23–24. 957044

[ref168] Miyazaki I and Harinasuta T (1966) The first case of human paragonimiasis caused by *Paragonimus heterotremus* Chen et Hsia, 1964. Annals of Tropical Medicine and Parasitology 60, 509–514.

[ref169] Miyazaki I and Ishii Y (1968) Studies on the Mexican lung flukes, with special reference to a description of *Paragonimus mexicanus* sp. nov. (Trematoda: Troglotrematidae). Japanese Journal of Parasitology 17, 445–453.

[ref170] Miyazaki I and Wykoff DE (1965) On a new lung fluke *Paragonimus siamensis* n. sp. found in Thailand (Trematoda: Troglotrematidae). Japanese Journal of Parasitology 14, 251–257.

[ref171] Mordvinov VA, Yurlova NI, Ogorodova LM and Katokhin AV (2012) *Opisthorchis felineus* and *Metorchis bilis* are the main agents of liver fluke infection of humans in Russia. Parasitology International 61, 25–31. 21840415 10.1016/j.parint.2011.07.021

[ref172] Nakamura-Uchiyama F, Mukae H and Nawa Y (2002) Paragonimiasis: a Japanese perspective. Clinics in Chest Medicine 23, 409–420. 12092035 10.1016/s0272-5231(01)00006-5

[ref173] Nakamura S (2017) Present situation of opisthorchiasis in Vientiane Capital, Lao Peoples’ Democratic Republic. Japanese Journal of Hygiene 72, 101–105 (in Japanese). 28552889 10.1265/jjh.72.101

[ref174] Narain K, Agatsuma T and Blair D (2010) Paragonimus. In Liu D (ed.), Molecular Detection of Foodborne Pathogens. Boca Raton, USA: CRC Press, Taylor & Francis Group, pp. 827–837.

[ref175] Nawa Y, Doanh PN and Thaenkham U (2013) Is *Opisthorchis viverrini* an avian liver fluke? Journal of Helminthology 89, 255–256. 24160690 10.1017/S0022149X13000709

[ref176] Nkouawa A, Okamoto M, Mabou AK, Edinga E, Yamasaki H, Sako Y, Nakao M, Nakaya K, Blair D, Agatsuma T, Enyong P, Shibahara T, Moyou-Somo R and Ito A (2009) Paragonimiasis in Cameroon: molecular identification, serodiagnosis and clinical manifestations. Transactions of the Royal Society of Tropical Medicine and Hygiene 103, 255–261. 18992906 10.1016/j.trstmh.2008.09.014

[ref177] Oh SJ (1968 *a*) *Paragonimus* meningitis. Journal of the Neurological Sciences 6, 419–433. 5304520 10.1016/0022-510x(68)90028-2

[ref178] Oh SJ (1968 *b*) Spinal paragonimiasis. Journal of the Neurological Sciences 6, 125–140. 5644938 10.1016/0022-510x(68)90130-5

[ref179] Oh SJ (1969) Cerebral and spinal paragonimiasis. A histopathological study. Journal of the Neurological Sciences 9, 205–236. 5345111 10.1016/0022-510x(69)90072-0

[ref180] Pakharukova MY and Mordvinov VA (2016) The liver fluke *Opisthorchis felineus*: biology, epidemiology and carcinogenic potential. Transactions of the Royal Society of Tropical Medicine and Hygiene 110, 28–36. 26740360 10.1093/trstmh/trv085

[ref181] Parkinson M, Dalton JP and O'Neill SM (2011) Fasciolosis. In Palmer SR, Soulsby L, Torgerson P and Brown DWG (eds), Oxford Textbook of Zoonoses (2nd ed.). Biology, Clinical Practice, and Public Health Control. Oxford, UK: Oxford University Press, pp. 862–872.

[ref182] Pozio E, Armigbacco O, Ferri F and Morales MAG (2013) *Opisthorchis felineus*, an emerging infection in Italy and its implication for the European Union. Acta Tropica 126, 54–62. 23337391 10.1016/j.actatropica.2013.01.005

[ref183] Procop GW (2009) North American paragonimiasis (caused by *Paragonimus kellicoti*) in the context of global paragonimiasis. Clinical Microbiology Reviews 22, 415–446. 19597007 10.1128/CMR.00005-08 PMC2708389

[ref184] Pungpak S, Viravan C, Radomyos B, Chalermrut K, Yemput C, Plooksawasdi W, Ho M, Harinasuta C and Bunnag D (1997) *Opisthorchis viverrini* infection in Thailand: studies on the morbidity of the infection and resolution following praziquantel treatment. American Journal of Tropical Medicine and Hygiene 56, 311–314. 9129534 10.4269/ajtmh.1997.56.311

[ref185] Rabone M, Wiethase J, Clark PF, Rollinson D, Cumberlidge N and Emery AM (2021) Endemicity of *Paragonimus* and paragonimiasis in Sub-Saharan Africa: a systematic review and mapping reveals stability of transmission in endemic foci for a multi-host parasite system. PLoS Neglected Tropical Diseases 15, e0009120. 33544705 10.1371/journal.pntd.0009120 PMC7891758

[ref186] Raghunathan VS and Srinivasan T (1962) *Artyfechinostomum mehrai* infestation. A case report. Journal of Indian Medical Association 38, 485–487. 14489877

[ref187] Rajapakse RPVJ, Pham KLT, Karunathilake KJK, Lawton SP and Le TH (2020) Characterization and phylogenetic properties of the complete mitochondrial genome of *Fascioloides jacksoni* (syn. *Fasciola jaksoni*) support the suggested intergeneric change from *Fasciola* to *Fascioloides* (Platyhelminthes: Trematoda: Plagiorchiida). Infection, Genetics and Evolution 82, 104281. 10.1016/j.meegid.2020.104281 32165245

[ref188] Rim HJ (1982 *a*) Clonorchiasis. In Steele JH, Hillyer GV and Hopla CE (eds), CRC Handbook Series in Zoonoses, Section C: Parasitic Zoonoses, Vol. III. Trematode Zoonoses. Boca Raton, FL, USA: CRC Press, pp. 17–32.

[ref189] Rim HJ (1982 *b*) Opisthorchiasis. In Steele JH, Hillyer GV and Hopla CE (eds), CRC Handbook Series in Zoonoses, Section C: Parasitic Zoonoses, Vol. III. Trematode Zoonoses. Boca Raton, FL, USA: CRC Press, pp. 109–121.

[ref190] Rim HJ (1982 *c*) Echinostomiasis. In Steele JH, Hillyer GV and Hopla CE (eds), CRC Handbook Series in Zoonoses, Section C: Parasitic Zoonoses, Vol. III. Trematode Zoonoses. Boca Raton, FL, USA: CRC Press, pp. 53–69.

[ref191] Rim HJ (2005) Clonorchiasis: an update. Journal of Helminthology 79, 269–281. 16153321 10.1079/joh2005300

[ref192] Rim HJ, Chai JY, Min DY, Cho SY, Eom KS, Hong SJ, Sohn WM, Yong TS, Deodato G, Standgaard H, Phommasack B, Yun CH and Hoang EH (2003) Prevalence of intestinal parasite infections on a national scale among primary schoolchildren in Laos. Parasitology Research 91, 267–272. 14574555 10.1007/s00436-003-0963-x

[ref193] Romero-Alvarez D, Valverde-Muñoz G, Calvopina M, Rojas M, Cevallos W, Kumazawa H, Takagi H and Sugiyama H (2020) Liver fluke infections by *Amphimerus* sp. (Digenea: Opisthorchiidae) in definitive and fish intermediate hosts in Manabí province, Ecuador. PLoS Neglected Tropical Diseases 14, e0008286. 32598382 10.1371/journal.pntd.0008286 PMC7351216

[ref194] Rosa BA, Choi YJ, McNulty SN, Jung H, Martin J, Agatsuma T, Sugiyama H, Le TH, Doanh PN, Maleewong W, Blair D, Brindley PJ, Fischer PU and Mitreva M (2020) Comparative genomics and transcriptomes of 4 *Paragonimus* species provide insights into lung fluke parasitism and pathogenesis. GigaScience 9, 1–16. 10.1093/gigascience/giaa073 PMC7370270 32687148

[ref195] Roy B and Tandon V (1992) *Opisthorchis noverca* Braun, 1902: first record from a bovine host and a comparative stereoscan study of the surface topography of flukes of swine and cattle origin. Acta Parasitologica 37, 179–181.

[ref196] Saijuntha W, Sithithaworn P, Kiasopit N, Andrews RH and Petney TN (2019) Liver flukes: Clonorchis and Opisthorchis. In Toledo R and Fried B (eds), Digenetic Trematodes. Advances in Experimental Medicine and Biology, vol. 1154. pp. 139–180. Available at 10.1007/978-3-030-18616-6_6. 31297762

[ref197] Saijuntha W, Sithithaworn P, Petney TN and Andrews RH (2021) Foodborne zoonotic parasites of the family Opisthorchiidae. Research in Veterinary Science 135, 404–411. 33158552 10.1016/j.rvsc.2020.10.024

[ref198] Saito S, Chai JY, Kim KH, Lee SH and Rim HJ (1997) *Metagonimus miyatai* sp. nov. (Digenea: Heterophyidae), a new intestinal trematode transmitted by freshwater fishes in Japan and Korea. Korean Journal of Parasitology 35, 223–232. 9446902 10.3347/kjp.1997.35.4.223

[ref199] Sanpool O, Intapan PM, Thanchomnang T, Janwan P, Nawa Y, Blair D and Maleewong W (2013) Molecular variation in the *Paragonimus heterotremus* complex in Thailand and Myanmar. Korean Journal of Parasitology 51, 677–681. 24516273 10.3347/kjp.2013.51.6.677 PMC3916457

[ref200] Seo BS (1990) *Fibricola seoulensis* Seo, Rim and Lee, 1964 (Trematoda) and fibricoliasis in man. Seoul Journal of Medicine 31, 61–96.

[ref201] Seo BS, Hong ST and Chai JY (1981) Studies on intestinal trematodes in Korea III. Natural human infection of *Pygidiopsis summa* and *Heterophyes heterophyes nocens*. Seoul Journal of Medicine 22, 228–235.

[ref202] Seo M, Guk SM, Kim J, Chai JY, Bok GD, Park SS, Oh CS, Kim MJ, Yi YS, Shin MH, Kang IU and Shin DH (2007) Paleoparasitological report on the stool from a medieval child mummy in Yangju, Korea. Journal of Parasitology 93, 589–592. 17626351 10.1645/GE-905R3.1

[ref203] Shan X, Lin C, Li Y, Hu Y, Shen X and Lou H (2009) A new species of *Paragonimus sheni*-with a key to the species of adult worms and metacercariae of the genus *Paragonimus* in China. Chinese Journal of Zoonoses 25, 1143–1148.

[ref204] Sherrad-Smith E, Cable J and Chadwick EA (2009) Distribution of Eurasian otter biliary parasites, *Pseudamphistomum truncatum* and *Metorchis albidus* (Family Opisthorchiidae), in England and Wales. Parasitology 136, 1015–1022. 19523253 10.1017/S0031182009006362

[ref205] Shibahara T, Nishida H, Torii M, Gyoten J, Tsuboi T and Sakai M (1992) Experimental infection of wild boars with metacercariae of *Paragonimus miyazakii* (Trematoda: Troglotrematidae). Japanese Journal of Parasitology 41, 274–278.

[ref206] Shin EH, Im TK, Park YK, Cho J, Kim JL and Chai JY (2016) Fecundity reduction of BALB/c mice after survival from lethal *Neodiplostomum seoulense* infection. Parasitology Research 115, 2051–2059. 26857130 10.1007/s00436-016-4949-x

[ref207] Shoriki T, Ichigawa-Seki M, Suganuma K, Naito I, Hayashi K, Nakao M, Aita J, Mohanta UK, Inoue N, Murakami K and Itagaki T (2016) Novel methods for the molecular discrimination of *Fasciola* spp. on the basis of nuclear protein-coding genes. Parasitology International 65, 180–183. 26680160 10.1016/j.parint.2015.12.002

[ref208] Shu QH, Li SD, Tian M, Meng Y, He SMQ, Wang MM and Wang WL (2021 *a*) Morphological and molecular characterization of *Paragonimus skrjabini* complex from Yunnan, China: a brief report. Acta Parasitologica 67, 316–321. 10.1007/s11686-021-00461-w 34417714 PMC8938381

[ref209] Shu QH, Yang Y, Wang MM, Li SD, Tian M, Bai WW, Meng Y, He SMQ and Wang WL (2021 *b*) Morphological and molecular characterization of *Paragonimus* species isolated from freshwater crabs in southern Yunnan, China. Journal of Tropical Medicine 2021, 5646291. 35003270 10.1155/2021/5646291 PMC8741392

[ref210] Shumenko PG, Tatanova YV and Besprozvannykh VV (2017) *Metagonimus suifunensis* sp. n. (Trematoda: Heterophyidae) from the Russian Southern Far East: morphology, life cycle, and molecular data. Parasitology International 66, 982–991. 27836745 10.1016/j.parint.2016.11.002

[ref211] Simpson VR, Gibbons LM, Khalil LF and Williams JLR (2005) Cholecystitis in otters (*Lutra lutra*) and mink (*Mustela vison*) caused by the fluke *Pseudamphistomum truncatum*. Veterinary Record 157, 49–52. 16006641 10.1136/vr.157.2.49

[ref212] Singh ST, Singh DL and Sugiyama H (2006) Possible discovery of Chinese lung fluke, *Paragonimus skrjabini* in Manipur, India. Southeast Asian Journal of Tropical Medicine and Public Health 37(suppl.), 53–56. 17547053

[ref213] Singh TS, Sugiyama H, Umehara A, Hiese S and Khalo K (2009) *Paragonimus heterotremus* infection in Nagaland: a new focus of paragonimiasis in India. Indian Journal of Medical Microbiology 27, 123–127. 19384034 10.4103/0255-0857.49424

[ref214] Singh TS, Sugiyama H, Devi KR and Singh WA (2015) First case of *Paragonimus westermani* infection in a female patient in India. Indian Journal of Medical Microbiology 33(suppl. 1), S156–S159. 10.4103/0255-0857.150950 25657140

[ref215] Sithithaworn P and Haswell-Elkins M (2003) Epidemiology of *Opisthorchis viverrini*. Acta Tropica 88, 187–194. 14611873 10.1016/j.actatropica.2003.02.001

[ref216] Sitko J, Bizos J, Sherrad-Smith E, Stanton DWG, Komorova P and Heneberg P (2016) Integrative taxonomy of European parasitic flatworms of the genus *Metorchis* Looss, 1899 (Trematoda: Opisthorchiidae). Parasitology International 65, 258–267. 26794684 10.1016/j.parint.2016.01.011

[ref217] Skrjabin KJ and Podjapolskaja WP (1931) *Nanophyetus schikhobalowi* n. sp., ein neuer Trematode aus dem Darm des Menschen. Zentralblatt für Bakteriologie und Parasitenkunde (Orig.) 119, 294–297 (in Germany).

[ref218] Slepchenko S (2020) *Opisthorchis felineus* as the basis for the reconstruction of migrations using archaeparasitogical materials. Journal of Archaeological Science: Reports 33, 102548.

[ref219] Sohn WM, Kim HJ, Yong TS, Eom KS, Jeong HG, Kim JK, Kang AR, Kim MR, Park JM, Ji SH, Sinuon M, Socheat D and Chai JY (2011) *Echinostoma ilocanum* infection in Oddar Meanchey Province, Cambodia. Korean Journal of Parasitology 49, 187–190. 21738278 10.3347/kjp.2011.49.2.187 PMC3121079

[ref220] Sohn WM, Yong TS, Eom KS, Min DY, Lee D, Jung BK, Banouvong V, Insisiengmay B, Phammasack B, Rim HJ and Chai JY (2014) Prevalence of *Haplorchis taichui* among humans and fish in Luang Prabang Province, Lao PDR. Acta Tropica 136, 74–80. 24754919 10.1016/j.actatropica.2014.04.020

[ref221] Sohn WM, Yong TS, Eom KS, Sinuon M, Jeoung WG and Chai JY (2017) *Artyfechinostomum malayanum*: metacercariae encysted in *Pila* sp. snails purchased from Phnom Penh, Cambodia. Korean Journal of Parasitology 55, 341–345. 28719961 10.3347/kjp.2017.55.3.341 PMC5523902

[ref222] Sohn WM, Jung BK, Hong SJ, Lee KH, Park HB, Kim HS, Cho S, Htoon TT, Tin HH and Chai JY (2019) Low-grade endemicity of opisthorchiasis, Yangon, Myanmar. Emerging Infectious Diseases 25, 1435–1437. 31211941 10.3201/eid2507.190495 PMC6590760

[ref223] Sripa B, Kaewkes S, Intapan PM, Maleewong W and Brindley PJ (2010) Food-borne trematodiases in Southeast Asia: epidemiology, pathology, clinical manifestations and control. Advances in Parasitology 72, 305–349. 20624536 10.1016/S0065-308X(10)72011-X

[ref224] Sripa B, Brindley PJ, Mulvenna J, Laha T, Smout MJ, Mairiang E, Bethony JM and Loukas A (2012) The tumorigenic liver fluke *Opisthorchis viverrini*-multiple pathways to cancer. Trends in Parasitology 28, 395–407. 22947297 10.1016/j.pt.2012.07.006 PMC3682777

[ref225] Sripa B, Tangkawattana S and Brindley PJ (2018) Update on pathogenesis of opisthorchiais and cholangiocarcinoma. Advances in Parasitology 102, 97–113. 30442312 10.1016/bs.apar.2018.10.001

[ref226] Steverding D (2020) The spreading of parasites by human migratory activities. Virulence 11, 1177–1191. 32862777 10.1080/21505594.2020.1809963 PMC7549983

[ref227] Sugiyama H, Morishima Y, Binchai S, Rangsiruji A and Ketudat P (2007) New form of *Paragonimus westermani* discovered in Thailand: morphological characteristics and host susceptibility. Southeast Asian Journal of Tropical Medicine and Public Health 38, 87–91.

[ref228] Sugiyama H, Singh TS and Rangsiruji A (2013) Paragonimus. In Liu D (ed.), Molecular Detection of Human Parasitic Pathogens. Boca Raton, USA: CRC Press, Taylor & Francis Group, pp. 423–435.

[ref229] Tandon V, Prasad PK, Chatterjee A and Bhutia PT (2007) Surface fine topography and PCR-based determination of metacercaria of *Paragonimus* sp. from edible crabs in Arunachal Pradesh, Northeast India. Parasitology Research 102, 21–28. 17786478 10.1007/s00436-007-0715-4

[ref230] Tandon V, Roy B and Prasad PK (2013) Fasciolopsis. In Liu D (ed.), Molecular Detection of Human Parasitic Pathogens. Boca Raton, USA: CRC Press, Taylor & Francis Group, pp. 353–364.

[ref231] Tang M, Zhou Y, Liu Y, Cheng N, Zhang J and Xu X (2021) Molecular identification and genetic-polymorphism analysis of *Fasciola* flukes in Dali Prefecture, Yunnan Province, China. Parasitology International 85, 102416. 34217794 10.1016/j.parint.2021.102416

[ref232] Tantrawatpan C, Tapdara S, Agatsuma T, Sanpool O, Intapan PM, Maleewong W and Saijuntha W (2021) Genetic differentiation of Southeast Asian *Paragonimus* Braun, 1899 (Digenea: Paragonimidae) and genetic variation in the *Paragonimus heterotremus* complex examined by nuclear DNA sequences. Infection, Genetics and Evolution 90, 104761. 10.1016/j.meegid.2021.104761 33577999

[ref233] Teimoori S, Arimatsu Y, Laha T, Kaewkes S, Sereerak P, Sripa M, Tangkawattana S, Brindley PJ and Sripa B (2017) Chicken IgY-based coproantigen capture ELISA for diagnosis of human opisthorchiasis. Parasitology International 66, 443–447. 27140305 10.1016/j.parint.2015.10.011 PMC5086311

[ref234] Terasaki K, Habe S, Ho LY, Jian H, Agatsuma T, Shibahara T, Sugiyama H and Kawashima K (1995) Tetraploid of the lung fluke *Paragonimus westermani* found in China. Parasitology Research 81, 627–630. 7479656 10.1007/BF00932031

[ref235] Thaenkham U, Nuamthanong S, Vonghachck Y, Yoonuan T, Sanguankiat S, Dekumyoy P, Phommasack B, Kobayashi J and Waikagul J (2011) Discovery of *Opisthorchis lobatus* (Trematoda: Opisthorchiidae): a new record of small liver flukes in the Great Mekong Sub-region. Journal of Parasitology 97, 1152–1158. 21682557 10.1645/GE-2764.1

[ref236] Thaenkham U, Blair D, Nawa Y and Waikagul J (2012) Families Opisthorchiidae and Heterophyidae: are they distinct? Parasitology International 61, 90–93. 21740979 10.1016/j.parint.2011.06.004

[ref237] Thatcher VE (1970) The genus *Amphimerus* Barker, 1911 (Trematoda: Opisthorchiidae) in Colombia with the description of a new species. Proceedings of the Helminthological Society of Washington 37, 207–211.

[ref238] Tran BT, Nguyen ST, Nguyen TT, Luc PV, Mafie E, Rupa FH and Sato H (2016) Endoparasites of Vietnamese lizards recorded in the last 50 years (1966–2015). Japanese Journal of Veterinary Parasitology 15, 34–58.

[ref239] Traversa D, Lorusso V and Otranto D (2013) Dicrocoelium. In Liu D (ed.), Molecular Detection of Human Parasitic Pathogens. Boca Raton, London, New York: CRC Press Taylor & Francis Group, pp. 323–331.

[ref240] Urabe M (2003) Trematode fauna of prosobranch snails of the genus *Semisulcospira* in Lake Biwa and the connected drainage system. Parasitology International 52, 21–34. 12543144 10.1016/s1383-5769(02)00083-1

[ref241] Vo DT, Murrell D, Dalsgaard A, Bristow G, Nguyen DH, Bui TN and Vo DT (2008) Prevalence of zoonotic metacercariae in two species of groupers, *Epinephelus coioides* and *Epinephelus bleekeri*, and flathead mullet, *Mugil cephalus*, in Vietnam. Korean Journal of Parasitology 46, 77–82. 18552542 10.3347/kjp.2008.46.2.77 PMC2532609

[ref242] Waikagul J (2007) A new species of *Paragonimus* (Trematoda: Troglotrematidae) from a cat infected with metacercariae from mountain crabs *Larnaudia larnaudii*. Journal of Parasitology 93, 1496–1500. 18314698 10.1645/GE-1054.1

[ref243] Waikagul J, Wongsaroj T, Radomyos P, Meesomboon V, Praewanich R and Jongsuksuntikul P (1997) Human infection of *Centrocestus caninus* in Thailand. Southeast Asian Journal of Tropical Medicine and Public Health 28, 831–835. 9656410

[ref244] Wang W, Blair D, Min T, Li F and Wang D (2011) *Paragonimus* worm from a New Guinea native in 1926. Asian Pacific Journal of Tropical Medicine 4, 76–78. 21771422 10.1016/S1995-7645(11)60038-2

[ref245] Wanlop A, Wongsawad C, Prattapong P, Wongsawad P, Chontananarth T and Chai JY (2017) Prevalence of *Centrocestus formosanus* metacercariae in ornamental fish from Chiang Mai, Thailand, with molecular approach using ITS2. Korean Journal of Parasitology 55, 445–449. 28877579 10.3347/kjp.2017.55.4.445 PMC5594729

[ref246] Witenberg G (1929) Studies on the trematode-family Heterophyidae. Annals of Tropical Medicine and Parasitology 23, 131–268.

[ref247] Witenberg G (1932) On the anatomy and systematic position of the causative agent of so-called salmon poisoning. Journal of Parasitology 18, 258–263.

[ref248] World Health Organization (1995) Control of foodborne trematode infections. Report of a WHO Study Group. WHO Technical Report Series 849, 1–158. 7740791

[ref249] World Health Organization (2020) Ending the Neglect to Attain the Sustainable Development Goals: A Road Map for Neglected Tropical Diseases 2021–2030. Geneva: World Health Organization, pp. 1–196. Available at https://www.who.int/publications/i/item/9789240010352.

[ref250] Wykoff DE, Harinasuta C, Juttijudata P and Winn MM (1965) *Opisthorchis viverrini* in Thailand – the life cycle and comparison with *O. felineus*. Journal of Parasitology 51, 207–214. 14275209

[ref251] Xiao X, Wang T, Zheng X, Shen G and Tian Z (2005) In vivo and in vitro encystment of *Echinochasmus liliputanus* cercariae and biological activity of the metacercariae. Journal of Parasitology 91, 492–498. 16108537 10.1645/GE-445R

[ref252] Yaemput S, Dekumyoy P and Visiassuk K (1994) The natural first intermediate host of *Paragonimus siamensis* (Miyazaki and Wykoff, 1965) in Thailand. Southeast Asian Journal of Tropical Medicine and Public Health 25, 284–290. 7855641

[ref253] Yamaguti S (1958) Part I. The digenetic trematodes of vertebrates. In Systema Helminthum, vol. I. New York, USA: Interscience Publishers Inc., pp. 1–1074.

[ref254] Yamaguti S (1971) Synopsis of Digenetic Trematodes of Vertebrates. Vols. I (pp. 1–1074) & II (349 Figure Plates). Tokyo, Japan: Keigaku Publishing Co.

[ref255] Yang B, Li J and Zhou B (2021) Analysis of internal transcribed spacer regions II gene and morphology of *Paragonimus* from Yunnan Province, China. Research Square 2021, 1–19. 10.21203/rs.3.rs-699536/v1

[ref256] Yatera K, Hanaka M, Hanaka T, Yamasaki K, Nishida C, Kawanami T, Kawanami Y, Ishimoto H, Kanazawa T and Mukae H (2015) A rare case of paragonimiasis miyazakii with lung involvement diagnosed 7 years after infection: a case report and literature review. Parasitology International 64, 274–280. 10.1016/j.parint.2015.02.009 25771073

[ref257] Ye HY and Mitchell PD (2016) Ancient human parasites in ethnic Chinese populations. Korean Journal of Parasitology 54, 565–572. 27853113 10.3347/kjp.2016.54.5.565 PMC5127531

[ref258] Yokogawa M (1965) *Paragonimus* and paragonimiasis. Advances in Parasitology 3, 99–158. 5334823 10.1016/s0065-308x(08)60364-4

[ref259] Yokogawa M, Harinasuta C and Charoenlarp P (1965) *Hypoderaeum conoideum* (Block, 1782) Dietz, 1909, a common intestinal fluke of man in the north-east Thailand. Japanese Journal of Parasitology 14, 148–153.

[ref260] Yokogawa M, Araki K, Saito K, Momose T, Kimura M, Suzuki S, Chiba N, Kutsumi H and Minai M (1974) *Paragonimus miyazakii* infections in man first found in Kanto district, Japan. Japanese Journal of Parasitology 23, 167–179.

[ref261] Yong TS, Chai JY, Sohn WM, Eom KS, Jeoung HG, Hoang EH, Yoon CH, Jung BK, Lee SH, Sinuon M and Socheat D (2014) Prevalence of intestinal helminths among inhabitants of Cambodia (2006–2011). Korean Journal of Parasitology 52, 661–666. 25548418 10.3347/kjp.2014.52.6.661 PMC4277029

[ref262] Yoshida A, Doanh PN and Maruyama H (2019) *Paragonimus* and paragonimiasis in Asia: an update. Acta Tropica 199, 105074. 31295431 10.1016/j.actatropica.2019.105074

[ref263] Youssef MM, Mansour NS, Awadalla HN, Hammouda NA, Khalifa R and Boulos LM (1987 *a*) Heterophyid parasites of man from Idku, Maryut and Manzala Lakes areas in Egypt. Journal of the Egyptian Society of Parasitology 17, 475–479. 3693948

[ref264] Youssef MM, Mansour NS, Hammouda NA, Awadalla HN, Khalifa R and Boulos LM (1987 *b*) Studies on some developmental stages in the life cycle of *Pygidiopsis genata* Looss, 1907 (Trematoda: Heterophyidae) from Egypt. Journal of the Egyptian Society of Parasitology 17, 463–474. 3693947

[ref265] Yu JR and Chai JY (2013) Metagonimus. In Liu D (ed.), Molecular Detection of Human Parasitic Pathogens. Boca Raton, USA: CRC Press, Taylor, Francis Group, pp. 389–398.

[ref266] Yu SH and Mott KE (1994) Epidemiology and morbidity of food-borne intestinal trematode infections. Tropical Diseases Bulletin 91, R125–R152.

[ref267] Yu S, Zhang X, Chen W, Zheng H, Ai G, Ye N and Wang Y (2017) Development of an immunodiagnosis method using recombinant PsCP for detection of *Paragonimus skrjabini* infection in humans. Parasitology Research 116, 377–385. 27796563 10.1007/s00436-016-5300-2

[ref268] Zhou XJ, Yang Q, Tan QH, Zhang LY, Shi LB and Zhou JX (2021) *Paragonimus* and its host in China: an update. Acta Tropica 223, 106094. 34389330 10.1016/j.actatropica.2021.106094

