# Supplementary material for: General overview of the current status of human foodborne trematodiasis
Source: Parasitology. 2022 May 20;149(10):1262–85. doi: 10.1017/S0031182022000725 (PMC10090779; doi:10.1017/S0031182022000725)
Supplement: Supplementary file 1 [file S0031182022000725sup001.docx]

**Supplementary Table.** Miscellaneous intestinal fluke species^a^ with their life cycle and geographical distribution

| Group | Species | First intermediate host | Second intermediate host | Reservoir host | Geographical distribution^b^ |
| --- | --- | --- | --- | --- | --- |
| Heterophyids | *Acanthotrema felis* | unknown | brackish water fish  (*Acanthogobius flavimanus*) | cat | Korea |
|  | *Apophallus donicus* | stream snail  (*Flumenicola virens*) | freshwater fish  (coho salmon, blackside dace, suckers) | dog, cat, rat, fox, rabbit | USA |
|  | *Ascocotyle longa* | cochliopid snail  (*Heleobia australis*) | brackish water fish  (*Mugil liza*) | dog | Canada, Europe, Ukraine, USA |
|  | *Centrocestus armatus* | freshwater snail (*Semisulcospira libertina*) | freshwater fish  (*Zacco platypus*) | dog, cat, rabbit, rat, bird | Korea, Japan |
|  | *Centrocestus cuspidatus* | freshwater snail (*Oncomelania formosana*) | freshwater fish  (*Gambusia* sp.) | dog, cat, fox, rat, kite, chicken | China, Egypt, Kuwait, Taiwan, Tunisia |
|  | *Centrocestus kurokawai* | unknown | freshwater fish (?) | unknown | Japan |
|  | *Cryptocotyle lingua* | *Littorina* snails  (*Littorina littorea*) | freshwater and brackish water fish  (*Gobius ruthensparri*) | dog, cat, rat, fox, gull, tern, heron | Denmark, England, Greenland, Iceland, Japan, North America, Norway, Russia, Ukraine |
|  | *Haplorchis vanissimus* | unknown | freshwater fish (?) | cormorant, eagle, heron, dog, cat | Australia, The Philippines |
|  | *Heterophyes dispar* | brackish water snail (*Pirenella conica*) | brackish water fish  (*Mugil* spp.) | dog, cat, fox, jackal, fox, wolf, kite | Egypt, Middle East |
|  | *Metagonimus katsuradai* | freshwater snail (*Semisulcospira libertine*) | freshwater fish  (*Tanakia* spp*.*) | dog (experimentally cat, dog, rat, rabbit, hamster) | Japan, Russia |
|  | *Metagonimus minutus* | unknown | brackish water fish  (*Mugil cephalus*) | experimental cat, mouse | Taiwan |
|  | *Procerovum calderoni* | brackish water snail (*Sermyla riquetti*) | freshwater fish  (*Ophiocephalus striatus*) | dog, cat | China, Egypt, The Philippines |
|  | *Procerovum varium* | brackish water snail (*Melanoides tuberculata*) | brackish water fish  (*Mugil affinis*) | dog (exp.), cat | Australia, Cambodia, China, Egypt, India, Japan, Korea, Laos, The Philippines, Vietnam |
|  | *Stictodora lari* | brackish water gastropod (*Velacumantus australis*) | esturine fish, goby | cat, dog (exp.) | Japan, Korea, Australia |
| Echinostomes | *Echinochasmus jiufoensis* | freshwater snail (?) | snail, fish (?) | dog, cat, pig | China |
|  | *Echinoparyphium recurvatum* | planorbid or lymnaeid snail (*Physa* sp., *Lymnaea* spp.) | snail, tadpole, frog | dog, house rat, wild rat | Bangladesh, Bulgaria, Canada, China, Croatia, Czech Republic, Egypt, England, India, Indonesia, Japan, Korea, Mexico, The Philippines, New Zealand, Poland, Russia, Spain, Taiwan, Thailand, USA |
|  | *Echinostoma aegyptica* | unknown | unknown | rat | China, Egypt, Japan, Taiwan, Turkey, Vietnam |
|  | *Echinostoma angustitestis* | freshwater snail (?) | freshwater fish | dog, livestock | China |
|  | *Echinostoma macrorchis* | freshwater snail (*Segmentina hemisphaerula*) | freshwater snail  (*Segmentina hemisphaerula*), tadpole, frog | rat, mouse, bird | Japan, Korea, Laos, Taiwan |
|  | *Echinostoma paraensei* | freshwater snail (*Biomphalaria glabrata*) | freshwater snail (*Biomphalaria glabrata*) | rat | Australia, Brazil |
|  | *Himasthla muehlensi* | marine gastropod (?) | marine gastropod, bivalve, annelid (?) | bird, mammal (?) | North America (USA) |
|  | *Isthmiophora melis* | freshwater snail  (*Lymnaea stagnalis*) | amphibians, fish | rodent, fox, marten, badger, hedgehog, mink | Belarus, Bulgaria, Canada, Czech Republic, England, France, Germany, Hungary, Lithuania, Poland, Romania, Russia, Taiwan, Ukraine, USA |
| Other group | *Cotylurus japonicus* | freshwater snail (?) | freshwater snail (?) | duck | China, India, Japan, Korea (South), Russia |
|  | *Fibricola cratera* | freshwater snail  (*Physa gyrina*) | frog, tadpole | muskrat, brown rat, mouse, shrew | Canada, USA |
|  | *Fischoederius elongatus* | freshwater snail  (*Lymnaea luteola*) | aquatic plants | ruminants (cattle, buffalo, caribous, goat, sheep) | China, England, India, Indonesia, Japan, Russia, Sri Lanka, Taiwan, Thailand, Vietnam |
|  | *Gynaecotyla squatarolae* | brackish water snail  (*Batillaria cumingi*) | brackish water crab  (*Macrophthalmus dilatatus*) | shorebirds  (*Squatarola squatarola*) | Japan, Korea (South), Taiwan |
|  | *Isoparorchis hypselobagri* | prosobranch snail  (*Melanoides tuberculatus*) | fish (catfish) | siluroid or non-siluroid fish | Australia, Bangladesh, China, India, Indonesia, Japan, Pakistan, Russia, Thailand, Vietnam |
|  | *Phaneropsolus spinicirrus* | unknown | naiad of dragonfly (?) | not found (only human) | Thailand |
|  | *Plagiorchis harinasutai* | freshwater snail (?)  (*Lymnaea* sp.) | snail, insect larva, fish (?) | rodent, bat, bird (?) | Thailand |
|  | *Plagiorchis javensis* | freshwater snail (?) | snail, insect larva, fish (?) | bat, bird | Indonesia |
|  | *Plagiorchis muris* | freshwater snail  (*Lymnaea pervia*) | snail, aquatic insect, shrimp, small fish | dog, cat, raccoon, bat, rat, mouse, bird | Cambodia, Central Europe, England, Iran, Ireland, Japan, Korea (South), Laos, Mexico, Netherlands, The Philippines, Spain, Taiwan, Thailand, USA, Vietnam |
|  | *Plagiorchis philippinensis* | unknown | insect larva (?) | rat, bird (?) | The Philippines |
|  | *Plagiorchis vespertilionis* | aquatic snail  (*Lymnaea stagnalis*) | insect larva | rodent, bat | Afghanistan, Belarus, Canada, China, Denmark, Egypt, France, Hungary, India, Iraq, Italy, Japan, Korea (South), Madagascar, Mexico, Mongolia, Poland, Romania, Russia, Spain, Taiwan, Turkey, Ukraine, USA |
|  | *Prohemistomum vivax* | freshwater snail (*Cleopatra bulimoides*) | brackish and freshwater fish | dog, cat, kite, rat (exp.) | Egypt, Israel, Europe |
|  | *Watsonius watsoni* | freshwater snail  (*Physa* sp*.*) | aquatic plants (?) | monkey, baboon | Nigeria, Senegal, Vietnam, Zambia |

^a^Miscellaneous intestinal flukes are defined as those having less than 10 reported human cases.

^b^The life cycles and geographical distributions of miscellaneous intestinal flukes are mostly referred from Chai (2019) and Chai and Jung (2019, 2020).
